# Between Order and Disorder: A ‘Weak Law’ on Recent Electoral Behavior among Urban Voters?

**DOI:** 10.1371/journal.pone.0039916

**Published:** 2012-07-25

**Authors:** Christian Borghesi, Jean Chiche, Jean-Pierre Nadal

**Affiliations:** 1 Centre d’Analyse et de Mathématique Sociales (CAMS), Centre National de la Recherche Scientifique et Ecole des Hautes Etudes en Sciences Sociales, Paris, France; 2 Laboratoire de Physique Théorique et Modélisation (LPTM), Centre National de la Recherche Scientifique et Université de Cergy-Pontoise, Cergy-Pontoise, France; 3 Centre de Recherches Politiques de Sciences Po (CEVIPOF), Centre National de la Recherche Scientifique et Sciences Po, Paris, France; 4 Laboratoire de Physique Statistique (LPS), Centre National de la Recherche Scientifique/École Normale Supérieure/Université Pierre et Marie Curie/Université Paris Diderot, Paris, France; University of Namur, Belgium

## Abstract

A new viewpoint on electoral involvement is proposed from the study of the statistics of the proportions of abstentionists, blank and null, and votes according to list of choices, in a large number of national elections in different countries. Considering 11 countries without compulsory voting (Austria, Canada, Czech Republic, France, Germany, Italy, Mexico, Poland, Romania, Spain, and Switzerland), a stylized fact emerges for the most populated cities when one computes the entropy associated to the three ratios, which we call the entropy of civic involvement of the electorate. The distribution of this entropy (over all elections and countries) appears to be sharply peaked near a common value. This almost common value is typically shared since the 1970s by electorates of the most populated municipalities, and this despite the wide disparities between voting systems and types of elections. Performing different statistical analyses, we notably show that this stylized fact reveals particular correlations between the blank/null votes and abstentionists ratios. We suggest that the existence of this hidden regularity, which we propose to coin as a ‘weak law on recent electoral behavior among urban voters’, reveals an emerging collective behavioral norm characteristic of urban citizen voting behavior in modern democracies. Analyzing exceptions to the rule provides insights into the conditions under which this normative behavior can be expected to occur.

## Introduction

Each election yields a variable proportion of citizens not taking part in the vote. The proportion of the uninvolved population – either by non-registering, abstaining or voting blank or null – has been much less studied than the vote itself.

Nowadays such behaviors are increasing among the longest-established democracies and their meaning may be changing. Besides passive abstention (due to carelessness or indifference), an active refusal of vote – possibly bearing a political message – is rising among population categories which are usually taking part in the election.

### The Modalities of Withdrawal [1]

To measure this phenomenon accurately, we first need to define the non-voter turnout. The boundary between voters and non-voters is indeed blurred as several intermediate behaviors exist, such as non-registering or blank vote.

The potential voter population depends on the legal requirements of citizenship, residency and capacity. Registration on the electoral roll does not necessarily imply voting. Moreover, the diversity of enumeration methods from one country to another makes it difficult to compare directly ratios of voters. The main trend consists in comparing abstention to the number of citizens entitled to vote (VEP: Voting Eligible Population). However, in the United States for instance, abstention was calculated until recently by comparison to the population above the voting age, including foreigners (VAP: Voting Age Population), the corresponding abstention rate often reaching 50%. Another bias stems from the fact that some countries made voting compulsory (namely Belgium, Luxembourg, Greece, and for a time the Netherlands, Austria and Italy). Without compulsory voting, a declining voter turnout is observed since the 1980s in established democracies.

Moreover, the meaning of blank and null vote is not obvious. They could be considered at first sight as equivalent to abstention or non-registering, since they seem to translate an absence of choice. This hypothesis would be in agreement with the systematic reviews of the minutes of polling stations for instance.

Abstention has been primarily considered to be a micro-level phenomenon. But is it really? Several studies have proven that socio-economic characteristics such as gender [2], [3], age [4], education [5], [6] and ethnicity [7] have an influence on electoral non-participation. To what extent does living in a community with low level of electoral involvement influence a voter?

### The Political and Institutional Context of the Election

The comparative database collected by the Institute for Democracy and Electoral Assistance (IDEA [8]) gathered data from elections in 171 countries from 1945 to 1999. It shows that participation rates are slightly higher in countries that have adopted a system of proportional representation, offering a larger choice to voters than those which have a majority or mixed systems. The highest turnout recorded (over 83% observed in both Malta and Ireland) corresponds to the system of ‘single transferable vote’ which gives the voter a large liberty margin. (This system, called Hare system of voting, is a variant of proportional representation where the voters rank the candidates according to their preferences.)

The nature of the election may be important too, depending on the context. In France for example, as the president has a lot of power, the participation rate of the presidential election is especially high when compared to the parliamentary election.

### Abstention and Blank and Null Votes

The reason why analysis of political sciences are paying little attention to blank and null votes is mostly based on the fact that these ballots are representing a very small number. Typically, these votes are aggregated within a single category, Blank and null votes, in some countries simply called Null (or Invalid) votes. Multitudinous studies have demonstrated from the 1950s on that null ballots were subdivided at random, according to the law of large number and distributed haphazardly for a given manner of voting [9]. The analysis of each voting office is still confirming that. However, the blank votes are more sensible to the conjuncture of consultation and are taking, with regard to abstention, a more complex signification.

Statistical analysis shows an often quite important negative correlation between abstention and Blank or invalid votes. In France, notably, it has often been observed that the more rural the municipality, the larger the ratio of Blank and null ballots. By contrast, the more populated the city, the larger the abstention ratio. However, the link between Abstention and Blank and null ballots becomes more complex in urban context. The urbanization has led to important changes in lifestyle and therefore in the voting behavior in large municipalities. Voters casting a blank vote are having motivations closer to voters abstaining for political reasons. This “civic abstention”, as Alain Lancelot called it, expresses a particular attitude regarding the voting procedure [9], [10]. This political attitude of “withdrawal” or political “offside” is not easy to analyze.

### Looking for Stylized Facts

In this paper, we analyze electoral data in order to better understand the interrelation between Abstention, Blank and null ballots and the expression of the vote, focusing on highly populated municipalities and recent elections. For this aim, we consider together the three values: Abstention, Blank/null and Valid votes ratios. We identify statistical regularities with an approach in the spirit of recent statistical physics analysis of elections data – see e.g. [11]–[23].

By analyzing a large number of elections in 11 different countries without compulsory voting, we point out that they share a common feature when considering highly populated municipalities in recent elections (as specified later). Introducing a measure of civic involvement of electorate, we show that this quantity exhibits a sharply peaked distribution around a common value. Moreover we suggest that this common stylized fact, that we propose to call a ‘weak law on recent electoral behavior among urban voters’, reveals an emerging collective behavioral norm, typical of citizen voting behavior in modern democracies.

The paper is organized as follows. First we describe the dataset used in this study, at three different scales (at the municipality scale, at larger scale but for older times, and at the polling station level when it is possible). Then, we introduce and discuss what we call the involvement entropy. We then analysis electoral data according to this measure, and give signs of existence of a possible norm revealed by a common-value of this measure. The Appendix S1 gives more details when it is necessary.

## Materials and Methods

### Dataset

In this paper we analyze electoral data at three different scales. (1) Data aggregated at the municipality scale. By this way, we study phenomena with respect to the population size of municipalities. The 76 elections studied in this paper at municipality level are mostly recent, after 1990, and are taken from 11 different countries (Austria, Canada, Czech Republic, France, Germany, Italy, Mexico, Poland, Romania, Spain and Switzerland). (2) Electoral data aggregated at large scale, e.g. national, provincial, etc. Here, we focus the analysis on time evolution. Countries studied for their historical aspects are those which are studied at the municipality scale. The study begins at the earliest year as possible, i.e. at the beginning of so-called democratic regimes, after World War II, and even earlier for some cases (e.g. 1884 for the 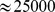 Swiss referendums). (3) Electoral data aggregated at the polling station level. Polling stations over the 100 most populated municipalities are analyzed, whenever it is possible to do so (i.e. for Canada, France, Mexico, Poland and Romania). Some intra-towns phenomena are investigated by this way.

Some elections are studied as a function of the number 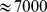 of registered voters by municipality. This is the case when the following conditions are valid: (1) elections in a democratic country with no compulsory voting, and no duty against people who do not vote; (2) the number of registered voters by municipality is well established (in particular this excludes from our study both the U.S.A. and England); (3) available data provide for each municipality, at least, the number of registered voters, the number of votes or the turnout rate, and the number of valid votes. We note that all countries for which we have the data at the municipality scale have more than 2000 municipalities, which allows us to make statistical analysis. Moreover, all elections studied here are national ones, except for *Land* Parliament elections in Germany. Lastly, the choice of the studied elections is not rooted on a plan but simply on the availability of electoral data.

Among these 76 elections, 31 of them are also analyzed at polling station level in the 100 most populated town: 5 from Canada (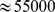 polling stations), 13 from France (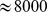 polling stations), 4 from Mexico (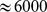 ballot boxes), 11 from Poland (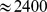 polling stations), and 4 from Romania (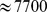 polling stations). Tab.1 summarizes the set of elections studied in this paper, and more details on these data are given in Appendix S1, Section A.

**Table 1:** Countries where elections are analyzed in this paper (first column).

At	13	1945	Ca*	5	1945	CH	3	1884	Cz	1	1990	Fr*	20	1946	Ge	7	1949
It	4	1946	Mx*	4	1991	Pl*	11	1990	Ro*	4	1990	Sp	4	1976			

Number of elections studied at the municipality scale (second column), and the date from which they are studied at national or provincial scale (third column) – even if it is before the end of the compulsory voting in Austria and in Italy. Star indicates that electoral data are also known at polling station level. Number of municipalities per country: 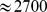 in Austria (At); 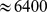 in Canada (Ca); 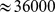 in Switzerland (CH); 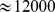 in Czech Republic (Cz); 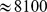 in Metropolitan France (Fr); 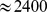 in Germany (Ge); 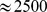 in Italy (It); 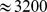 in Mexico (Mx); 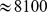 in Poland (Pl); 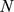 in Romania (Ro); 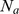 in Spain (Sp). See Appendix S1, Section A, for more details.

The Appendix S1, Section A, gives more information about the set of (public) electoral data studied in this paper. Most of them can be directly downloaded from official websites (see References in Appendix S1). Part of the database used in this paper can also be directly downloaded from [24].

### Abstentions, Valid Votes and Blank or Null Votes

**Figure 1:**
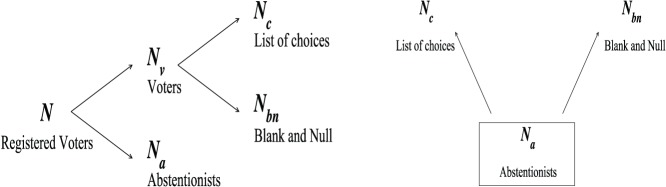
Electoral mobilization: categorization of registered voters according to their voting behavior. The latter may result from either two sequential binary decisions (left), or two mutually exclusive binary decisions (right), or (see Appendix S1, Section D.2, for a discussion).

Let us describe the citizen classification here retained to characterize the electoral mobilization of registered voters. For each given election and each specific scale (a municipality, a province, a country, etc.) we distinguish: (1) the total number 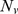 of registered Voters; (2) the number 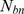 of Abstentionists, the persons who do not take part to the election; (3) the number 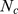 of voters, among which (4) 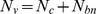 Blank and Null Votes (some countries, like Canada and Poland, aggregate Blank and Null votes in an only one term called as Null votes, or Invalid votes, or Spoilt votes) and (5) 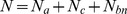 Votes in favor of candidates or electoral list of choices, also sometimes called Valid Votes (see Fig. 1). Obviously 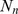 and 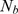. Note that in Italy, Spain, and Switzerland, electoral data distinguish between Null Votes, 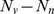, and Blank Votes, 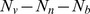. Moreover, only in Spain, *“Votos Válidos”* means 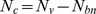, that differs from other countries where “Valid Votes” means 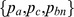. In this paper, we consider for all countries that Valid Votes are defined as 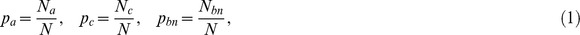. See Section F in Appendix S1 for more discussion about countries where Blank Votes and Null Votes are distinguished between each other.

**Figure 2:**
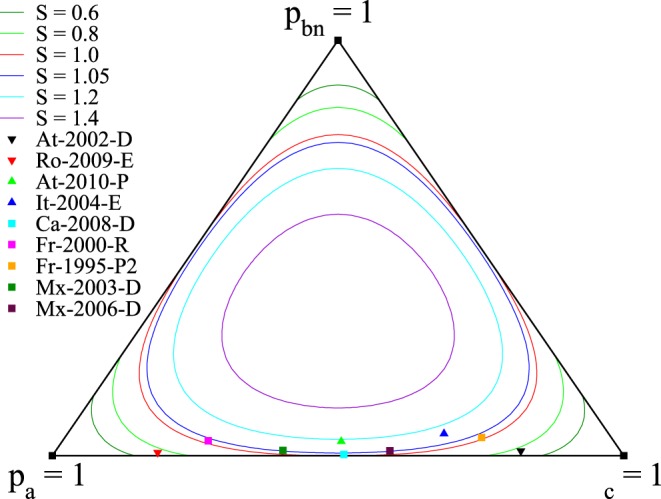
Simplex 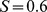, in which any given election, for the most populated municipalities, can be represented by a point, as illustrated by the symbols corresponding to particular elections of our data set (see Appendix S1, Section A, for details). The continuous curves are lines of constant involvement entropy value, drawn for values ranging from 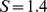 to 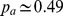. See text for At-2002-D, Ro-2009-E, At-2010-P and It-2004-E. For Ca-2008-D: 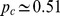, 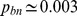, 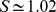 and 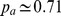; for Fr-2000-R: 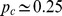, 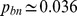, 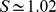 and 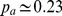; for Fr-1995-P2: 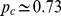, 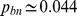, 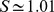 and 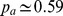; for Mx-2003-D: 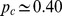, 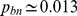, 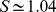 and 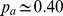; and for Mx-2006-D: 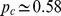, 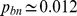, 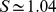 and 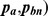.

**Figure 3:**
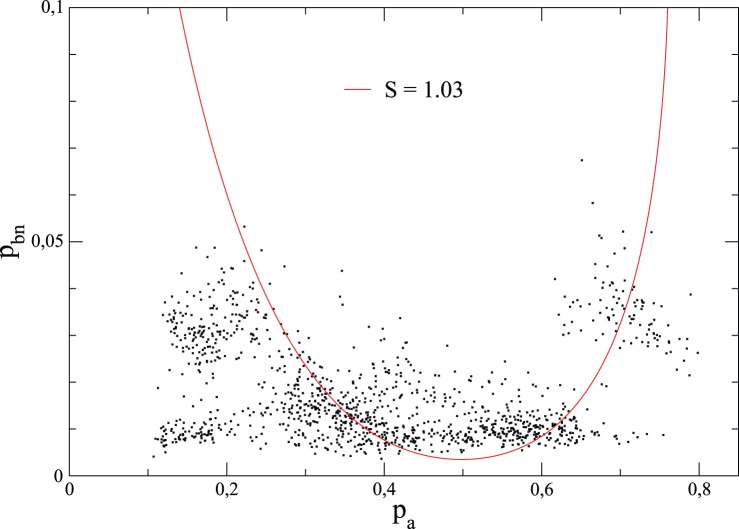
Scatter plot of (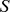 of the 100 most populated French cities over elections since 2000. The convex function 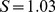 (here 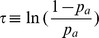) is also plotted as a guide view.

As discussed in the following, we characterize the civic involvement of registered voters by the choice between the three possible sates, Abstention, Blank or Null Vote and Valid Vote. The civic involvement of electors is then here measured through the set of the three ratios 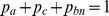, defined by


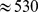


with 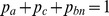. Each election can then be represented by a point in the simplex 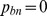, as illustrated on Fig. 2. Since the number of Blank and Null is typically small, clearly most points lie near the edge 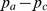. A second basic observation is that there is a wide dispersion along the axis 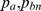. Figure 3 shows the scatter plot of (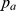) for French elections since 2000, and the 100 most populated cities. This plot suggests that individual behavior cannot be explained by a sequential binary choice (first to decide to vote or not, and if yes, then to decide to cast a valid vote or not), since this would lead to the absence of correlations between 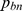 and 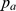. Hence the *electoral involvement* should be viewed through the three possibilities available to the voters: abstention, blank/null votes and votes according to the list of choices. Moreover, Figure 3 shows that, if there are statistical regularities, they can be seen by considering a convex function of the variables 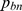, 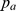. This is what we do below, making use of the entropy function associated to the three quantities 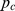, 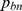 and 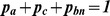.

Previous work [22] has revealed statistical regularities from election to election, and from country to country, when considering the distribution of turnout over municipalities. More precisely, the distribution of the logarithmic turnout rate, 
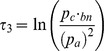
, *centered on its mean value*, is remarkably stable over time and across countries for the most populated cities. Similarly, a logarithmic three choices value can be defined, 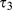, for which, the same type of regularities can be observed when considering polling stations within municipalities (see Appendix S1, Section D.1). In addition, this analysis of fluctuations confirms the remark in the preceding paragraph, that individual behavior is not well explained by a sequential binary choice (see Appendix S1, Section D.2). However, this analysis of fluctuations does not say anything on the mean values. In this paper, we exhibit another type of regularities, by considering an adequate function (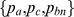 has not the appropriate convex properties), and focusing on the values themselves, not only the fluctuations around the means.

### The Involvement Entropy

We introduce a variable whose value, as we will argue, is appropriate for characterizing the mean civic involvement of the electorate. Viewing the three ratios 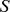 as probabilities, it is interesting to associate to each election, instead of these three numbers, a single scalar characterizing the probability distribution itself. One natural quantity associated to a probability distribution is the entropy, 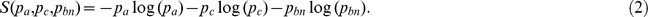, defined by


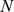


Here, and throughout this paper, 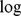 means base-two logarithm (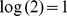, and the entropy is said to be in units of bits).

Within the framework of Information Theory, where it is called the Shannon entropy, this quantity can be understood as a measure of missing information, or of average surprise, associated to the studied random process [25]. In the context of Statistical Physics, it is the Boltzmann-Gibbs entropy measuring the degree of ‘disorder’ of the system under consideration [26]. In the present context, we will refer to 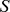 as the entropy of civic involvement, or “involvement entropy”, and consider it as a measure of disorder vs. order in the civic involvement at a collective level. Indeed, it is a ‘macroscopic’ or collective measure about the civic involvement of an electorate, and not the measure of the civic mobilization of individual citizen – i.e., we do not claim that it corresponds to the behavior of a representative citizen. It can be measured at any scale of aggregate data, e.g. for a municipality, a province, or a whole country. For instance, the involvement entropy of a municipality, 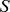, is given by Eq. (2) where the three ratios 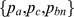 are the ratios of, respectively, the number abstentionists, 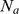, valid votes, 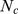, and blank and null votes 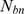, over the total number 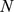 of registered voters in the considered municipality.

Let us explain more what we mean by ‘order/disorder’, and how this is reflected by the entropy value. We consider that a civic involvement shows an ‘ordered’ state if one of the three ratios is very close to one (hence the two others very small). A ‘disordered’ state corresponds to having all three ratios of similar values. Within this viewpoint, no particular role or importance is assigned to any one of the three possible cases, abstention, blank/null, valid vote. The involvement entropy 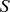, a positive or null quantity, provides a well defined way to quantify the degree of disorder: the larger the entropy, the larger the disorder. The maximum order is obtained when one of the ratios is equal to unity (and then the two others are equal to zero), in which case 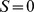. In contrast, the maximum disorder corresponds to an equipartition of these 3 ratios, that is 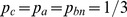, in which case the entropy takes its maximal possible value, 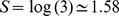.

**Figure 4:**
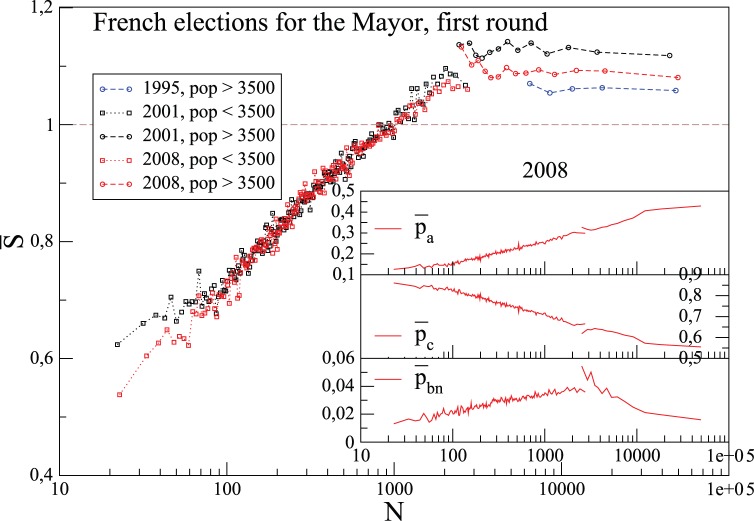
Average values 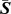 of the involvement entropy of municipalities, 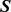, as a function of the number of registered voters 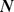, for the first round of Mayor elections in France. There are two kinds of voting rules, which depend on the population-size more or less than 3500 inhabitants (see text). Inset shows average values of 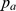, 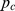 and 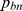 as a function of 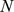 for the 2008 *municipales* elections (which lead for high population municipalities to a plateau of 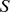 despite variations in 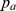, 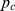 and 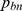). For each 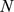, average values, 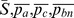, are evaluated over 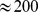 municipalities of size 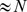.

As an illustration, consider the elections for the Mayor in the French municipalities. It is well known (at least in France) that participation to elections in small municipalities is typically larger than in large cities, for social reasons – for instance, in small municipalities where everyone knows every one else, not going to the polling station will become common knowledge. Such social enforcement of the civic involvement might be at the root of an increase of the number of abstentionists with population size: the ratio 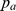 of abstentionists is typically very low for small municipalities, and increases with the municipality size, 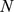. One then expects an increase of the involvement entropy with municipality-size: this is indeed what we observe for the elections for the 2001 and 2008 first round (elections for which we have the data for all the municipalities), as illustrated on Fig. 4. We can say that the electorate is very “ordered” (in terms of its civic involvement) for low municipality-size, and gets more “disordered” with increasing 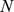. This involvement entropy increase is observed until a threshold population size value, at which the electoral rule changes: the citizen has a larger number of possible voting choices in municipalities with a number of inhabitants smaller than 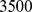, than in more populated municipalities. (It is allowed for citizens living in municipalities with less than 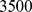 inhabitants, to combine candidates from different opposite lists, or to add new names from citizens who are not officially candidates.) Remarkably, above this critical size, the involvement entropy becomes essentially independent of the population size: one has a plateau, at 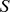 slightly above 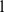, despite variations in 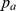, 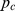 and 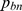. As we will see throughout this paper, this particular value of involvement entropy, 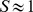, shows up as a typical value in modern elections for most populated cities.

**Figure 5:**
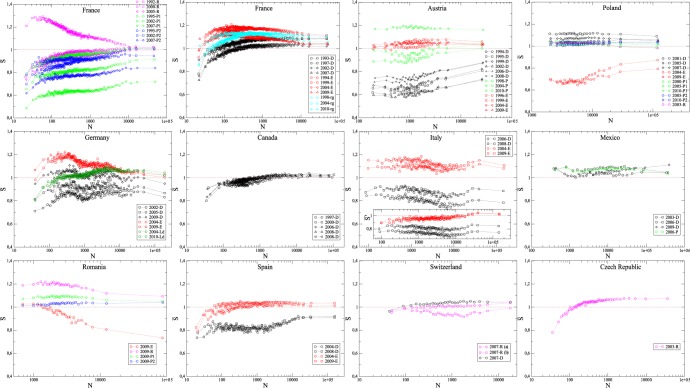
Mean values 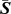 of the involvement entropy for municipalities, as a function of the number of registered voters 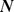. Each point results from an average over a sample of 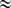 100 (200 for France) municipalities of size 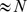. Italian graph inset shows a variant of 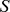 where Blank Votes are grouped with Valid Votes (see Appendix S1, Section F, for a deeper discussion). See the Appendix S1, Section A, for more details on the data.

Let us give other illustrations. A great order of the electorate is provided by: (1) the population of registered voters is highly polarized: there is an important difference between 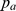 and 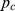 (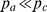 or 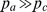); and (2) blank and/or null votes are very few, that is 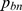 is very small. Such cases of small entropies are, e.g., the 2002 Austrian Chamber of Deputies election for which 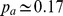, 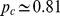, 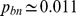 and 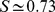; the 2009 European Parliament election in Romania, with 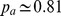, 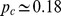, 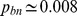 and 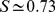. Conversely, a great disorder of the electorate results from: (1) the population of registered voters is not very polarized, that is 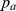 and 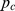 are not very different; and (2) blank and/or null votes are relatively important, that is 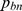 is not too small. For instance, the 2010 Austrian Presidential election has 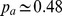, 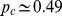, 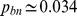 and 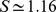; and the 2006 European Parliament election in Italy has 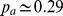, 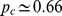, 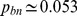 and 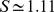. Note that these values come from great town values (see Tab. S1), whereas 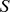 is more spread out in small municipalities (see Fig. 5). Finally, one finds that the involvement entropy 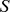 has a value frequently very near 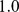. For example, the 2008 Canadian Chamber of deputies election, the 2000 French referendum, the 1995 French second round Presidential, and the 2003 and 2006 Mexican Chamber of deputies elections (see Fig. S2 and Tab. S1). In all these examples, despite an important diversity in 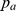 values, 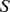 lies within 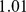 and 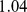, showing that the electorate polarization is somewhat halfway between order and disorder. Note that 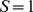 is the entropy associated to the tossing of a fair coin. In the present context, it would be exactly obtained for elections with 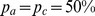 and 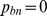.

## Results

### Stylized Fact: The Common Occurrence of 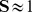


We have computed the involvement entropy 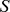 for all the elections of our data set, at different scales. First we find that, most often, it depends on the municipality-size 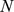. To analyze this size dependency, we spread out municipalities data over samples with respect to the municipality population-size. In each sample, municipalities have roughly the same number of registered voters. The number of municipalities per sample is of order 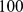, except for France in which case this number is 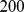 (because France has much more municipalities than the other countries studied in this paper). We denote by 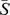 the average over all municipalities inside a sample of the involvement entropy 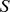. This average 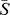 is plotted in Fig. 5 as a function of the number of registered voters, 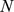.

In this paper, 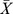 means the average value of the considered value, 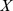, over all municipalities, around 100, or 200 for France, in a given sample where municipalities have roughly the same number of registered voters, 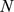; e.g. 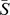, 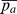, 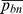, etc. Average values and standard-deviations do not take into account extreme values in order to remove some electoral errors, etc. Electoral values greater than *5 sigma* are not taken into account. For instance let 100 municipalities of size 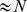 (as in Fig. 5), each one has a civic involvement entropy 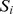 (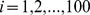). First, 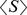 and 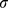 are the average value and the standard-deviation of 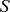 over these 100 municipalities. Next, the final average value 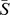 and the final standard-deviation over this sample of 100 municipalities are only evaluated for municipalities, 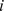, such that 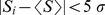.

Let us give the 1995 French second round Presidential election (Fr-1995-P2) as an example. A relatively ordered civic electorate involvement is observed for the smallest population-size municipalities, with 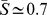. The mean involvement entropy then increases with municipality size, for sizes up to 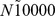. For the most populated municipalities, that is above this threshold value in population-size, a saturation occurs: the (average) civic disorder of the electorate becomes independent of municipality-size, with 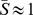.

**Figure 6:**
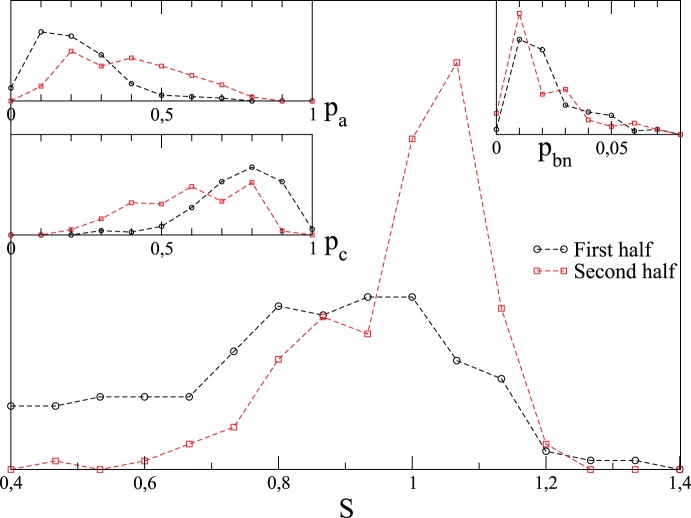
Evolution in time of involvement entropy, 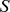, at large scale (national, provincial, etc.) of 321 elections (see Appendix S1, Section A, for more details), apart from Swiss referendums. For each country, electoral results are equally divided into two groups: those which occurred at the first period in time and at the second one. Histograms of 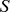 (and 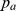, 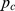 and 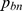 in the insets) show the involvement entropy of the first and second group over all countries. Fig. S1 plots for each country the whole of elections, and also Fig. S4 for scatter plots 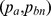 of these elections, but at national aggregate scale.

Next we consider the time evolution of the involvement entropy at a large scale (country, province, *canton*, etc.). When the scale of aggregate data is lower than the national one, each value of the involvement entropy for one election is equal to a weighted (by population-size) mean value of involvement entropies at lower scale (province, *canton*, etc.). (See Appendix S1, Section A, and Tab. S2 for more details.) Fig. S1 plots the involvement entropy of each election at large scale, for each country over all elections (according to its nature) as a function of time, and Fig. S2 shows how 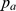 and 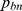 evolve in time for Chamber of Deputies election in each country. Nevertheless a rapid evolution in time of 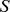 can be seen in a different way. First, for each country, elections are ordered according to their year; half of them, the more ancient ones, are gathered into one group, and the other half, the more recent ones, are gathered into another group. Next, we aggregate over countries, the “old” elections on one side, the “recent” ones on the other, getting a total of 321 elections split into two groups with roughly the same number of elections in each one. Although recency is here country specific, the aggregated group of recent elections corresponds more or less to those occurred since the 70s. The histograms of the involvement entropy 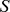 are compared for these two groups on Fig. 6. The histogram for the group of the more recent elections shows a sharp peak at 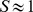, whereas the group of the older elections has a broad distribution. This temporal evolution occurs in parallel with a significant decrease of “highly ordered” elections (in the civic involvement point of view). In other words, nowadays there are few elections with a small civic involvement entropy, 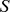 (say e.g. 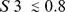), but there are a lot of elections with 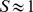.

**Figure 7:**
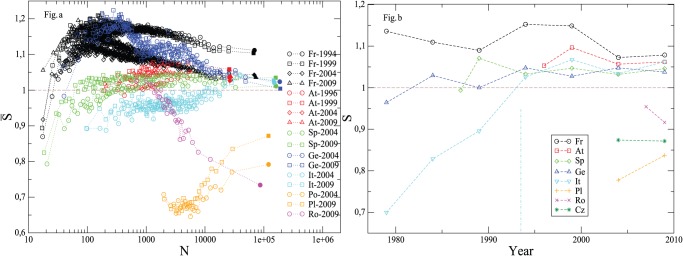
Mean involvement entropy for European Parliament elections. Fig. 7-a (left panel): same elections as those shown in Fig. 5; here for all countries, including France, averages are over 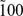 municipalities. Fig. 7-b (right panel): same elections as those shown in Fig. S1; the vertical dashed line indicates the year of the abolishment of compulsory voting in Italy. Here, Italian Blank Votes, 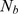, (but not Null Votes, 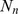) are grouped with votes in favor of lists of candidates (see Appendix S1, Section F, for more discussion).

Finally, Fig. 7 shows, for all the European Parliament Elections, how the involvement entropy of municipalities depends on population-size (like in Fig. 5), and the time evolution at the national or provincial scale (like Fig. S1).

### What the Common Occurrence is

**Figure 8:**
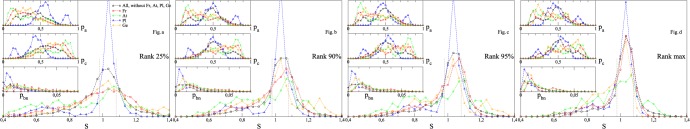
Histograms of involvement entropy, 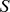, with respect to the relative municipality-size bin over all analyzed since 2000. There are 12 French elections, 7 Austrian elections, 11 Polish elections, 7 German elections and 24 for others countries (included in one curve, with no more than 4 elections per country). Municipalities of each country are divided into bins (of 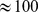 municipalities) with respect to their municipality-size (see e.g. Fig. 5). For instance, ‘Rank 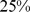
’ (Fig. 8-a) means the bin whose population-size rank is the twenty-fifth per cent with regard to the sample of the most populated municipalities (Fig. 8-d) of the considered country. Insets: histograms of corresponding 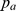, 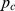 and 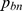. 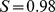 and 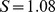 are plotted in dashed lines and all the scales axis are similar from one plot to another one.

**Figure 9:**
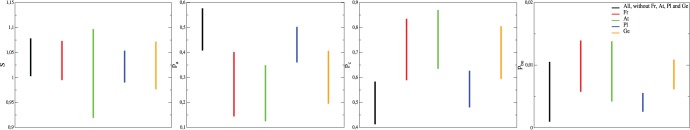
Minimal intervals containing 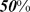 of events, of the involvement entropy 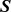 and ratios 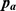, 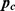 and 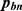 of the 100 most populated municipalities over all elections since 2000. See Fig. 8-d for the related histograms.

As already said, Fig. 5 shows the remarkable fact that, for each studied country, in modern elections the involvement entropy of highly populated municipalities is very frequently roughly equal to 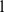. This common value, 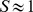, for high population-size municipalities is particularly striking when one looks at European Parliament Elections (see Fig. 7-a). See also Table 2 for a rapid overview and basic statistics per country about involvement entropies and population size of the 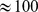 most populated municipalities. There are however noticeable exceptions, notably the Italian case on which we will come back later (Section Discussion). In any case, we have now to better specify what we mean by 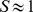 and show more quantitatively in which way it is a common property of modern elections. This is done by gathering data over all elections after 2000 (after 2000 in order to take into account evolution in time of the involvement entropy as stressed by Fig. 6 and Tab. 2). Fig. 8-d plots the resulting histograms of the involvement entropy restricted to 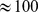 most populated municipalities, for different countries or ensemble of countries. Moreover, Fig. 9 shows respectively the minimal length interval of 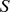, 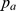, 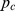 and 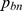 which contain 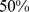 of events (those plotted in Fig. 8-d). These two figures show a common sharp peak at a value of 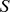 close to 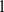. The involvement entropy appears to be mainly in the range 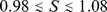, which can be taken as the definition of 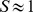 in this paper. Note that this definition is applied to the most populated municipalities. At large scale, the involvement entropy depends on the the way that data are aggregated (at national, province, etc. scale), and it is a little bit greater than 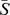 for the most populated municipalities. Nevertheless the involvement entropy measure at large scale approximately reflects how the most populated municipalities do, because an important ratio of population live in the 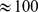 most populated municipalities (as seen in Tab. 2).

**Table 2:** Basic information about the bin of the 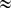
100 most populated municipalities per country (Ctry).

Ctry	date	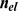	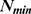	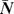	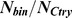	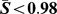	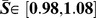	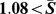
	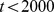							
Fr		8	32000	69000	18 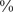	1	2	5
At		6	7000	26000	44 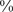	3	3	0
Ca		1	30000	94000	48 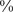	0	1	0
	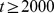							
Fr		12	33000	70000	17 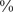	3	8	1
At		7	7000	27000	43 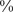	3	3	1
Pl		11	39000	120000	39 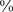	2	8	1
Ge		7	68000	190000	30 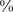	3	4	0
Ca		4	53000	83000	38 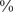	0	4	0
It		4	48000	150000	31 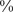	2	0	2
Sp		4	48000	160000	47 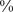	2	2	0
Mx		4	130000	370000	53 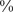	0	3	1
Ro		4	20000	87000	47 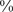	1	2	1
CH		3	7500	18000	37 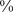	0	3	0
Cz		1	14000	36000	43 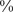	0	1	0

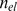
 means the number of elections analyzed. The municipality of this bin with the lowest number of registered voters is written as 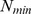; the average value of 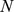 over these municipalities, as 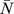; and the ratio of registered voters which belongs to this bin over those in the whole country, as 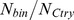. 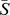 is classified according to values 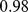 and 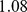.

It is important to stress that the common occurrence 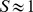 appears (1) as a property of high populated municipalities, (2) and also in a recent time. See Fig. 6, or Fig. S1, as an indication of the latter point. For the first point, Fig. 8 shows the histograms of the entropy for different municipality sizes. Compared with histograms of the most populated municipalities (Fig. 8-d), histograms of lower municipality-size appear: (1) much less peaked (apart from Polish elections), and (2) not peaked at the same common-value. Moreover, it is only for the larger sizes that all the histograms become very similar, suggesting the convergence to a universal histogram at large sizes. Let us bear in mind (cf. Tab. 2) that the sample of the 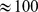 most populated municipalities in Austria is, on average, much less populated than the ones of the four other countries or ensemble or countries. (Taking into account the 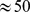 Austrian municipalities per sample provides, for the most populated sample, an histogram of 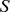 much centered on 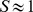 than the one of 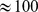 municipalities (see Fig. S3).) In other words, the Austrian sample of the 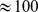 most populated municipalities is not so comparable to the four other ones, and does not accurately reflect a typical behavior in large populated municipalities (especially since the civic involvement can significantly depend on the population size as it is shown in Fig. 5). Lastly, the choice of the number (here 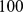) of most populated municipalities is only for statistical convenience and does not affect the results (see e.g. Fig. S3 which is similar to the Fig. 8-d, but for the sample of 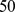 or 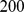 most populated municipalities).

**Figure 10:**
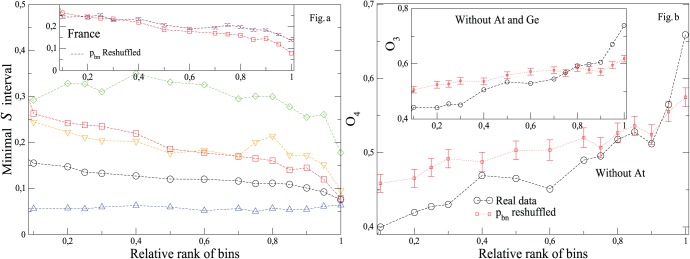
Quantitative evidence of the sharp and common peak of 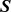 for the most populated municipalities. Considered elections, the way that bins are ranked, countries or groups of countries and legends are the same as in Fig. 8. Left (10-a): Minimal interval 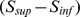, which encapsulates 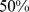 of events, with respect to the relative population size. Right (10-b): Overlap 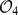 between 4 distributions of 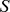 of municipalities as a function of their relative municipality-size (see text for the definition of 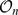). The inset shows in the same manner overlap 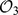 between 3 distributions of 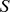 ((1): all without At, Fr, Ge and Pl; (2): Fr; (3): Pl). Some curves obtained from reshuffling 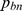 of municipalities (inside one country or ensemble of countries), while 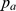 is not modified, are also plotted.

Now, let us better quantify this sharp and common peak for the most populated municipalities. First, Fig. 10-a plots the smallest distance 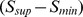, such that 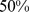 of events are included into the set 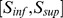, with respect to the relative municipality size. This confirms that (apart from Polish elections) distributions of 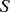 get more peaked when the population size increase, and specifically for the most populated municipalities. (The same features also appear by considering minimal distances which contain 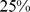 or 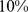 of events. This is in agreement of the robustness of this trivial method.) Moreover (apart from the Austrian elections) the minimal distance 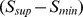 appears to converge to a common value, this only for the most populated sample (see also Fig. 9 for 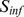 and 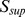 for this latter sample). Next, in order to quantify the common peak phenomenon, we calculate the overlap between distributions of 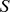 for municipalities as a function of the relative population size (see Fig. 10-b). The overlap between 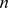 distributions of 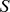, with probability density functions (pdf) 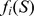, 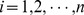, is defined as 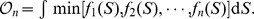 Fig. 10-b shows an increasing overlap between distributions when the population size increases, and specifically for the most populated municipalities. This confirms that the distributions of 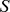 get more and more similar as the relative municipality-size increases, with (sharp) peaks becoming identical for the most populated municipalities.

### What the Common Occurrence is not

We claim that this common most frequent value, 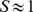 for the most populated municipalities, is not a mere statistical artefact. More precisely, we claim that:

1. vit is not a direct consequence of the law of large numbers, which, for data aggregated at the scale of large municipalities, would give a systematic result;
2. it is not a result of ‘pure chance’, that is a bias in the data due to random events, or an accidental bias in the collected data;
3. it does not only result from having 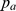 and 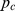 neither around 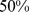 nor around a common value: there is a wide range of 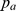 values for which 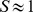 is observed;
4. it does not result from having a small proportion of Blank and Null Votes.

In support of the two first points, we show below that there are robust properties which cannot be explained by the pure chance or the large number hypotheses. About the two last points concerning the ranges of 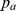 and 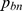 values, we show that, even if the distributions of 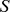 could be peaked for a relatively broad distribution of 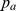 and small values of 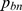, this, (1), cannot alone explain why the distributions of 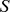 for the most populated towns are so much narrowed and, (2), in addition, have their peak at a common value of 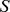. The next three sections detail these claims.

### Against a Randomness or Large Number Artefact. We note Three Facts that Goes Against a Pure Chance or Large Number Hypotheses

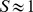
 is specific to modern elections. Indeed (apart from Swiss *Votations* discussed in Section Discussion) this common value 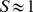 appears recently, and at different times for different countries – and different elections –: in the 70′s or 80′s in France, 80′s in Germany, 90′s in Canada, 2000’s in Czech Republic, etc (cf. Fig. S1). Moreover, there is no systematic way in which recent convergence to 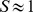 appears in time. 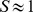 may be reached as well from inferior values (e.g. Chamber of Deputies elections in Canada, Czech Republic, etc., in Fig. 7-b) than from superior values (e.g. European Parliament in France in Fig. 7-b). Lastly, in a given country, some kind of elections provide at large scale 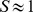 since their coming (e.g. European Parliament elections ), and for some other ones, 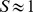 seems (actually) to be an attractor point in time (see e.g. Chamber of Deputies elections in Canada, Czech Republic, France, Switzerland, etc. in Fig. S1).

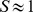
 is only observed for large populations (and there is no common-value for smaller municipality sizes) as it is shown in Fig. 10; and there is sometimes a plateau with a lower value of 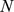 which both depend on the election and on the country (e.g. 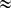 3000 in Canada and Czech Republic, 10000 in France for referendums, etc., in Fig. 5). Moreover, there is no systematic way in which convergence to 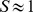 occurs as the population size increases. 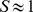 may be reached as well from inferior values (e.g. Fr-1995-P2, Sp-2004-E and Sp-2009-E) than from superior values (e.g. Fr-2000-R, Ge-2004-E and Ge-2009-E in Fig. 5). Lastly, 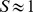 may be reached from a discontinuous transition when voting rule (which depends on the population size of municipalities) changes. This occurs for the two French local elections for the Mayor (see Fig. 4), which are the only one elections of our database where there is this electoral rule change.
- The shape of distributions of 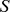 for large municipality sizes does not result from a statistical bias due to large numbers: creating artificial high populated municipalities, by means of aggregating large amount of citizen choices who live in small and different municipalities, does clearly not yield a distribution peaked near 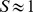 (see Appendix S1, Section C, and Fig. S6 for more details).

Finite-size-effects, that is the effect of aggregating data at different scales, are considered more thoroughly in Appendix S1, Section C, comparing ballot box scale with municipality scale. This section also discusses more the issue of statistical effects that could be due to large numbers.

#### Ranges of variation of 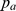 and 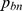


On one side, while 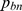 does not radically change in time at large scales, 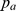 has increased during last decades in most countries (see e.g. insets of Fig. 6 and Fig. S2). On the other side, 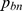 is known to decrease when the population-size of municipalities 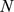 increases, as it was discussed in the Section Introduction. Let us thus first consider the possibility that the common occurrence 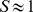 could be a consequence of these two facts: 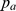 is not too small (for example, if 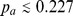, then it is no more possible to get 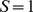) and, *independently*, 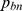 is small.

**Figure 11:**
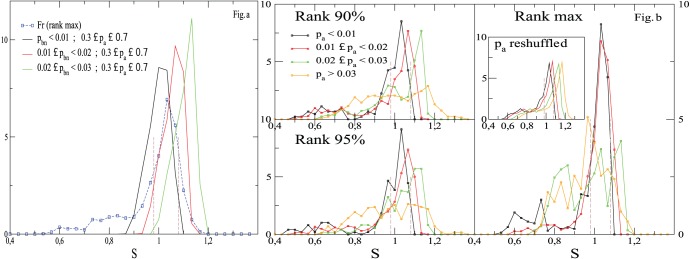
Relative importance of 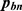 on the distribution of 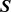. Left (11-a): Distribution of 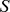 from flat distributions of 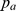 (where 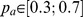) and 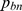. The pdf of 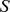 can be peaked for a relatively broad distribution of 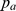 and a small range of 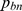, but the peak of the distribution of 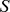, which depends on 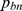 values, is not necessarily centered on 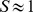. Histogram of 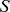 over the 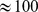 French most populated municipalities (the same as in Fig. 8-d) is also given as a guiding view. Right (11-b): pdf of 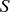, for different ranges of 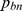, over 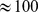 French municipalities, which depend on their relative population-size (like in Fig. 8-b, c, d). Histograms of 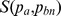 from reshuffled 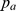, while 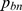 remain unchanged, are also given.

We give three arguments against this assertion. (i) First, we plot on Fig. 11-a histograms of 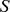 resulting from a flat and broad distribution of 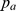, and a flat distributions of 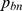 (with small values). Each histogram corresponds to a different choice of the range of (small) 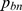 values. To better understand this point, let 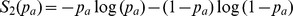 which has a maximal value, 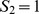, for 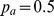. Moreover, when 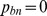, 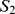 is equal to the involvement entropy, 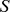 defined in Eq. (2), i.e. 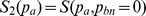. Hence, relatively small variations of 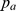 around 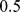 and very small values of 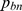 lead to 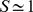.) The result is indeed a set of peaked histograms. However, these distributions of 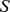 are neither necessarily centered on 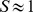 nor centered at a common peak.

(ii) Second, we emphasis the specificity of most populated municipalities. Fig. 11-b plots for French data (where the tested phenomenon is clearer) distributions of 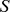 selecting elections for which 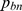 belongs to specific ranges of values. Moreover theses distributions are also plotted according to the population-size of municipalities. It is only for the most populated municipalities that the distributions of 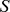 for different ranges of 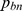 are roughly peaked at the same value 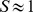 (with a very good agreement for 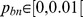 and 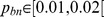). Moreover, for a lower population-size, e.g. with a relative rank of 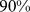, it is interesting to note that distributions of 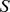 for different ranges of 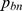 (apart from the 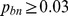) share the same features as in Fig. 11-a, i.e. distributions are peaked in different values. (To have a more detailed view, Fig. S5 shows scatter-plots 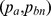 for the municipalities taken into account in Fig. 11-b.).

(iii) Third, there is actually a wide disparities in the ranges of 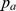 and 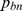 between different countries or group of countries. One can see in Fig. 9 how, (1), France and, (2), all countries without At, Fr, Ge and Pl, can reach the common 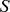 peak, despite largely different ranges of 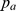, 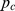 and 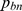. In other words, the ranges of ratios 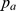, 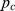 and 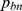 are not sufficiently similar between countries or ensemble of countries to explain why the distributions of 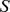 for the most populated municipalities share a sharp peak at a common value of 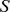.

#### Implied correlations between 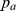 and 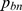


Hence, it seems difficult to explain the common value 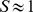 for the most populated towns as a consequence of having *independently* 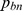 small and 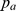 in a given particular range. The observation of a common peak around 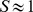 thus implies the existence of specific correlations between 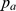 and 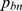.

To test this conclusion, we consider surrogate data obtained by reshuffling the ratios 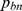 from one municipality (or country) to another one, while 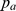 is kept unchanged (and then 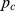 is deduced from 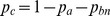). Note that the marginal distributions of 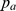 and 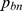 remain unchanged by this reshuffling procedure, whereas their correlations are destroyed. We use this method twice: first, (i) contrasting recent and old elections, and second, (ii), considering the dependency in municipality size. In the following, reshuffling results are shown as average values over 1000 realizations, and the corresponding standard-deviations are plotted as error bars.

**Figure 12:**
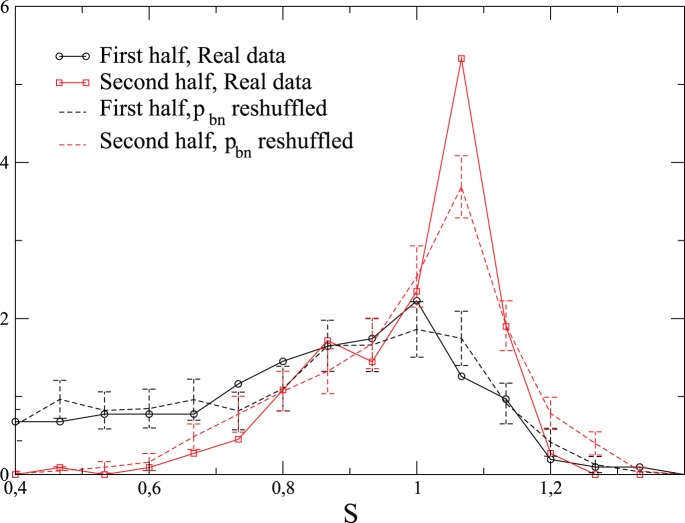
Relative importance of reshuffling 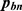 on 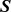. Analyzed elections and the manner that elections are divided into two groups are the same than in Fig. 6. Nevertheless, here, each election is aggregated at national scale, i.e. 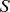 is directly evaluated from the set 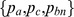 at the national scale. In these figures, surrogate 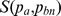 data, consist in reshuffling 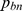 from one election to another one in the same group, while 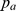 is not modified. Surrogate curves result from the average of 1000 realizations, and standard-deviations are plotted as error bars.

- Figure 12 shows, at national scale and for two periods of time, how the distributions of 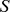 change under this reshuffling. 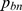 are reshuffled within the same group of elections. For the first period in time, the real distribution of 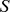, which is not peaked near 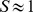, and the surrogate one are not very different between themselves. By contrast, the distributions are notably different for the second period. Moreover, the main difference concerns the peak near 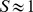. The peak of the surrogate data distribution is less sharp than the one of the real data. This is particularly interesting since 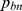 is roughly distributed in the same manner between the two relative periods in time (see insets of Fig. 6 or scatter-plots 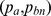 of Fig. S4). The widening of the surrogate distribution of involvement entropy near the peak 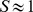 can be seen as a sign that there are correlations between 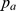 and 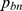 which enforces the occurrence of 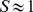.
- From a qualitative point of view, the reshuffled data have a peak of 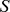 values which is less narrow than for the real ones, a discrepancy which increases with municipality size, as can be seen for the French data on the inset of Fig. 10-a, and on the scatter-plots 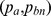 on Fig. S5. In addition, the distributions of 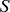 obtained for the reshuffled data are not as well peaked at a common value as it is the case for the real data ones. Quantitatively, for the French data, the Kolmogorov-Smirnov distance between the distributions of real and reshuffled data is significantly larger for the most populated municipalities, with a distance that allows one to reject the hypothesis that the two distributions are similar (indeed the Kolmogorov-Smirnov distance is then 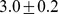, while 1.6 corresponds to 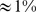 probability that the two distributions coincide). Moreover, Fig. 10-b shows that overlaps between different distributions of 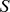 resulting from reshuffled 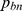 is smaller than for real data, and this only when municipality-sizes are high, or even only for the most populated municipality sample: the reshuffling suppresses the high increase of overlaps which is observed on real data for the sample of the most populated municipalities.

We can thus conclude that there is a specific property for the most populated municipalities, which is not encapsulated by considering 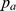 and 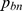 as independent variables.

## Discussion

We suggest that the common value 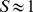 of the entropy, which appears recently in high populated municipalities, reveals an emerging collective behavioral norm characteristic of citizen involvement in modern democracies, and we propose to call it a ‘weak law’ on recent electoral behavior among urban voters. Signs of existence of this possible norm can not only be seen notably by the greatest density value of the involvement entropy 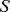 around 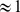, whatever countries, type of elections, etc., but also by its deviances. There are two kinds of deviances: for the fist one, 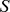 is small (which generally occurs when 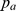 or 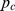 is very small), for the second one, 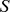 is high (which generally results from great ratio of blank or null votes, 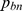). We will see that these deviances are associated with a particular phenomena of civic involvement, or are simply reduced to the norm (i.e 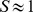) when the meaning of blank votes changes.

When significantly smaller values are observed (e.g. 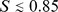) for cities, something appear inside towns (in average): the heterogeneity of involvement entropy over all polling stations of a given town decreases when 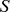 of the whole city decreases. In other words, considering the electorate civic involvement in a given town, the less is 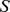 for the whole town, the more the town appears homogeneous (i.e. involvement entropies, at polling station scale, over all polling stations of the town are more homogeneous between themselves). Section E in Appendix S1 shows this point (free of statistical bias), particularly clear when the ratio 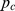 is high (compared to cases where 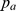 are high). This civic involvement phenomenon for towns with small 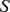 can be seen as a signature of something ‘new’ which appears when deviance of the norm occurs.

**Figure 13:**
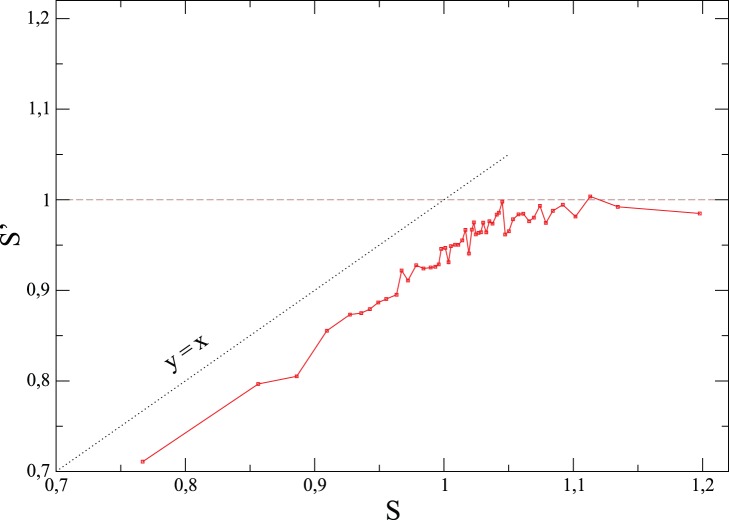
A modified involvement entropy, 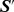, where Blank Votes are grouped with Valid Votes, with respect to the involvement entropy 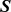, for 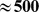 Swiss referendums. (See Appendix S1, Section F, for a deeper discussion.) Each point corresponds to the average of about 10 referendums. Note the plateau 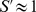 for 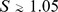.

On the other hand, elections where significantly 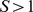, typically corresponds to cases where there has been an appeal (from political parties, citizens blogs, etc.) to vote blank or null, which adds civic-involvement ‘tensions’ to the election. It is remarkable that countries which make the distinction between blank votes to null votes, provide, by considering blank votes like the valid votes in favor of one of the list of choices, a modified involvement entropy 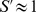 whenever the involvement entropy is 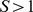. (When blank votes are grouped with votes according to the list of choices, the modified involvement entropy 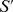 is equal to 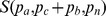 in Eq. (2), and not 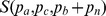 as for the usual involvement entropy, where 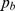, 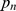 and 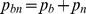 mean respectively ratios of blank votes, null votes and blank or null votes.) See the striking plateau in Fig. 13 for Swiss referendums, which shows a modified involvement entropy 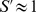 when 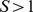. Moreover, Section F in Appendix S1 clearly shows this point, e.g. for European Parliament elections in Italy, and for Referendums in Spain. Hence the fact that 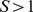 boils down to a modified involvement entropy 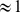, by categorizing blank votes as Valid Votes, can be seen as the recovering of the ‘weak law’ by the decrease of civic involvement ‘tensions’. The fact that a deviance of the norm is naturally reduced to the norm (the involvement entropy is around 1) as soon as blank votes are grouped with ‘valid votes’ can be seen not as an haphazardly occurrence but rather as a signature of the norm in a larger sense.

**Figure 14:**
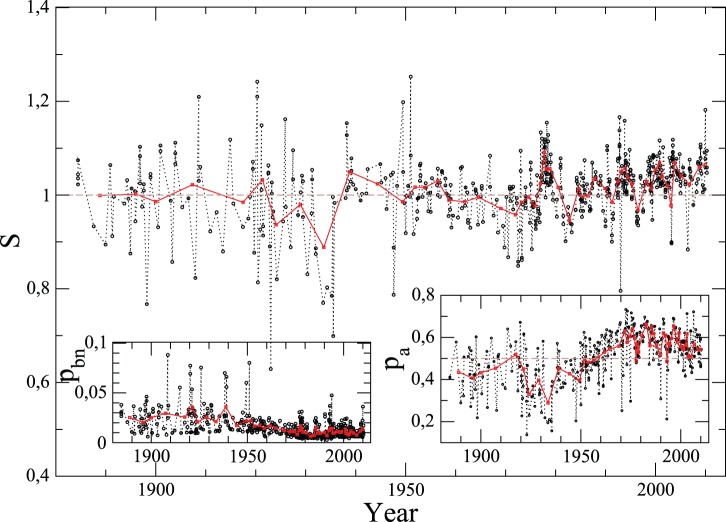
Time evolution of the involvement entropy, 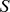, of 531 Swiss referendums, at large scale. Each point corresponds to the average (weighted by the number of registered voters) over all Swiss *cantons* (25 or 26 in quantity). In red (as a guide view): average values over 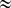
10 referendums. The inset show sames things, but for ratio of abstentionists, 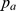, and the rartio of blank and null votes, 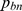.

Now let us comment about the use of the term ‘weak law’. In one hand, the common value 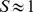 (for the most populated municipalities in recent times) appears as a kind of law of a phenomenon not yet measured up to now. This phenomenon concerns the involvement of the electorate, from a civic point of view. A kind of law, because it occurs very frequently, with strong regularities despite wide disparities across elections. As we have seen, it implies the existence of particular correlations between 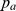 and 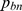. In other hand, this is clearly not a ‘hard’ or ‘strong’ law since noticeable deviations are observed. One cannot exclude that a ‘strong’ law exists, encapsulating more regularities for the most populated municipalities (e.g. by taking into account not only 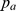, 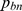 and 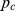, but also parameters characterizing the political context, the number of valid votes for different choices, etc.). Such more global law might explain why 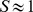 appears in recent times and why this phenomenon is not observed for small municipalities. In any case, we believe that this weak law of recent urban civic involvement shows up as a consequence of some robust electoral behavior. As one more illustration, Swiss referendums show (at the *canton* scale) 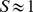 with small fluctuations, and this from 1880s to nowadays (see Fig. 14).

To conclude, the main finding of this work, based on the analysis of a wide number of elections from 11 different countries, is that a common stylized fact emerges: in recent elections, the distribution of the involvement entropy is found to be sharply peaked near 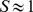, in high populated municipalities (and thus also at national levels). This universal property is remarkable given the wide disparities across countries (and even within countries for different elections) in political mores, voting systems, in the way that lists of registered voters are established (on a voluntary basis or automatically, etc.), and so on.

Moreover, 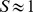 appears to be very stable in time whenever it occurs for one kind of election, as for example European Parliament elections in Western Europe, and particularly remarkably for the Swiss referendums since 1884. We propose to designate this strong regularity, neither a ‘hard law’ nor a mere statistical artefact, as a ‘weak law’ of electoral involvement characteristic of modern democracies in urban cities. We suggest that the existence of this weak law is the signature of an emerging collective behavioral norm. More studies and analysis would be necessary in order to better understand its conditions of realizations and its meaning (at the individual scale and/or at macro scale). Moreover, it should be very interesting for forthcoming studies, notably to know if this ‘weak law’ also occurs in emergent countries, in new democratic countries, in great cities (whatever they are), etc.

The present study calls for a different point of view than those commonly used in Political Sciences. We do not work within the classical paradigm explaining the electoral behavior with sociological or ethnic even institutional or rational choice variables. Our propose is to change perspective of observation, using very large sets of data, looking for regularities – stylized facts –, without restricting the analysis to a particular category which could be based on chronology, space, institutional or national specificities. At a ‘macro’ level, using aggregated data, and not at the individual scale, this new view point focuses on (1) the involvement or the mobilization of the electorate, and (2) a measure of heterogeneity or, otherwise stated, of order and disorder. The question asked here to electoral data is not why a more or less rational citizen participates or not to an election, but *how is the degree of disorder of civic involvement of the electorate*.

## Supporting Information

Figure S1 **Time evolution of the mean involvement entropy at large scale.** See Appendix S1, Section B, for more explanation.
(PDF)

Figure S2
**Moving average, as a function of time, per country of** 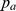 **and** 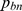 **at national scale for Chamber of Deputies elections.**
(PDF)

Figure S3
**Histograms of** 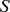 **for the** 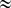 **200 and 50 most populated municipalities, similarly to** **Fig. 8** **-d (with 100 most populated municipalities for the latter one).**
(PDF)

Figure S4
**Evolution in time of scatter plots of** 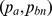 **at national level of 321 elections.**
(PDF)

Figure S5
**Scatter plots of** 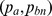 **of French municipalities according to their relative population size, over elections since 2000 (similarly as in** **Fig. 8-b, c, d)** **.**
(PDF)

Figure S6
**Histograms of** 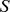 **of the 100 most populated towns compared with 100 artificial towns, in France, over elections since 2000.** See Appendix S1, Section C, for more explanation.(PDF)

Appendix S1A. Details on the data sources; B. More details on data analysis; C. Finite size effects; D. Logarithmic three choices value, 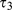, of polling stations; E. Looking for signs of tension, through polling stations analysis; F. Disentangling Blank votes from Null votes(PDF)

Table S1 Elections studied in this paper at the municipality scale. See Appendix S1, Section A, for more details.
(PDF)

Table S2 Elections studied in this paper at large scale (national, provincial, etc.) for their evolution in time. See Appendix S1, Section A, for more details.
(PDF)
