# Supplementary material for: Between Order and Disorder: A ‘Weak Law’ on Recent Electoral Behavior among Urban Voters?
Source: PLoS One. 2012 Jul 25;7(7):e39916. doi: 10.1371/journal.pone.0039916 (PMC3405122; doi:10.1371/journal.pone.0039916)
Supplement: Figure S1 — Time evolution of the mean involvement entropy at large scale. See Appendix S1, Section B, for more explanation. (PDF) [file pone.0039916.s001.pdf]

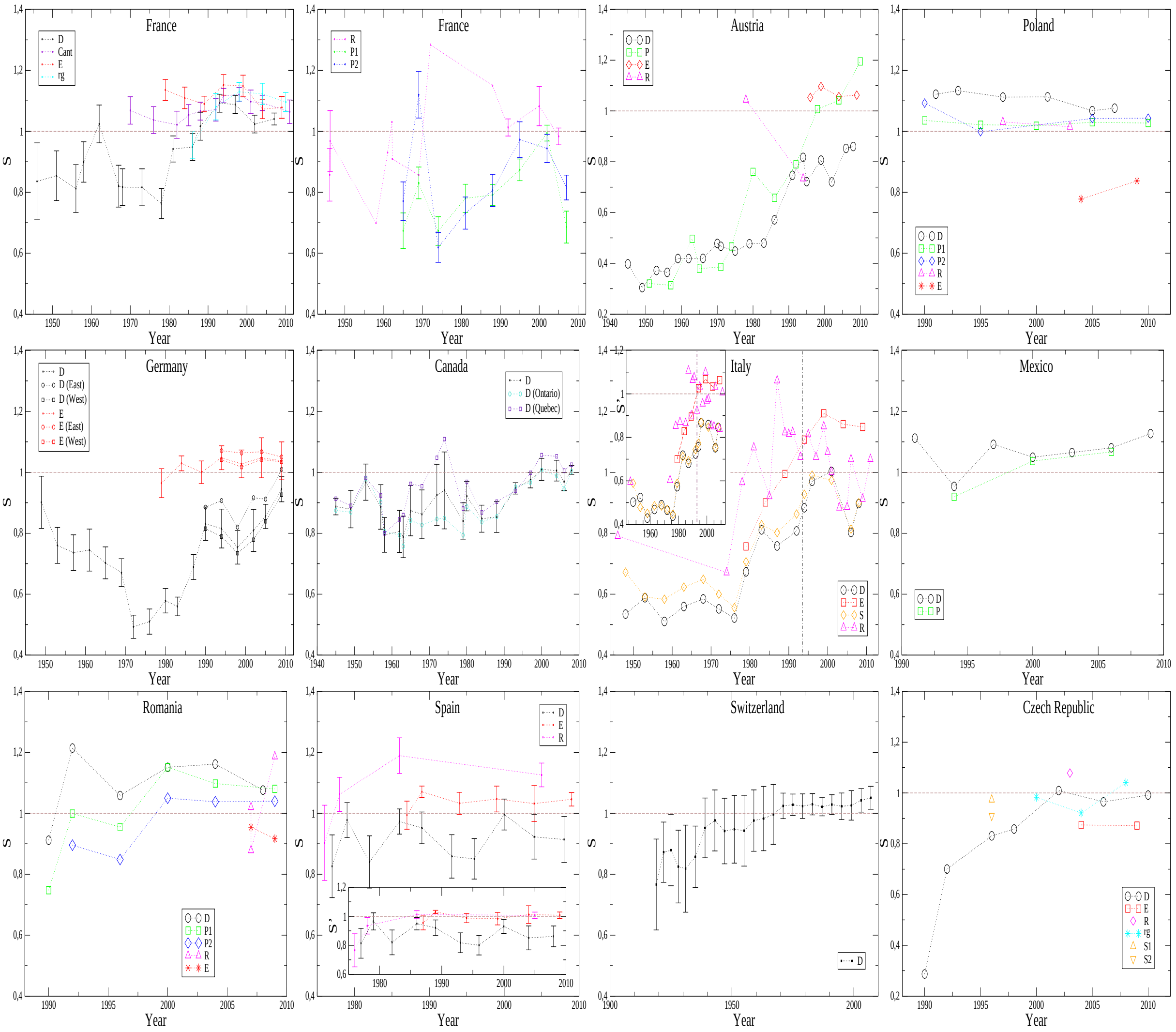

Figure S1: **Time evolution of the mean involvement entropy at large scale** (national, provincial, etc.). See Appendix S1, Section A and Tab. S2, for more details and also for the end of compulsory voting in Italy (cf. vertical dashed line) and in Austria. Whenever the scale of aggregate data is lower than the national one, standard-deviations (weighted by the number of registered voters) are also shown as error bars. Italian and Spanish graph insets show a variant of S where Blank Votes are categorized as Valid Votes (see Appendix S1, Section F, for more discussion). See Appendix S1, Section B, for more explanation about some French curves.
