# Supplementary material for: Between Order and Disorder: A ‘Weak Law’ on Recent Electoral Behavior among Urban Voters?
Source: PLoS One. 2012 Jul 25;7(7):e39916. doi: 10.1371/journal.pone.0039916 (PMC3405122; doi:10.1371/journal.pone.0039916)
Supplement: Figure S2 — Moving average, as a function of time, per country of and at national scale for Chamber of Deputies elections. (PDF) [file pone.0039916.s002.pdf]

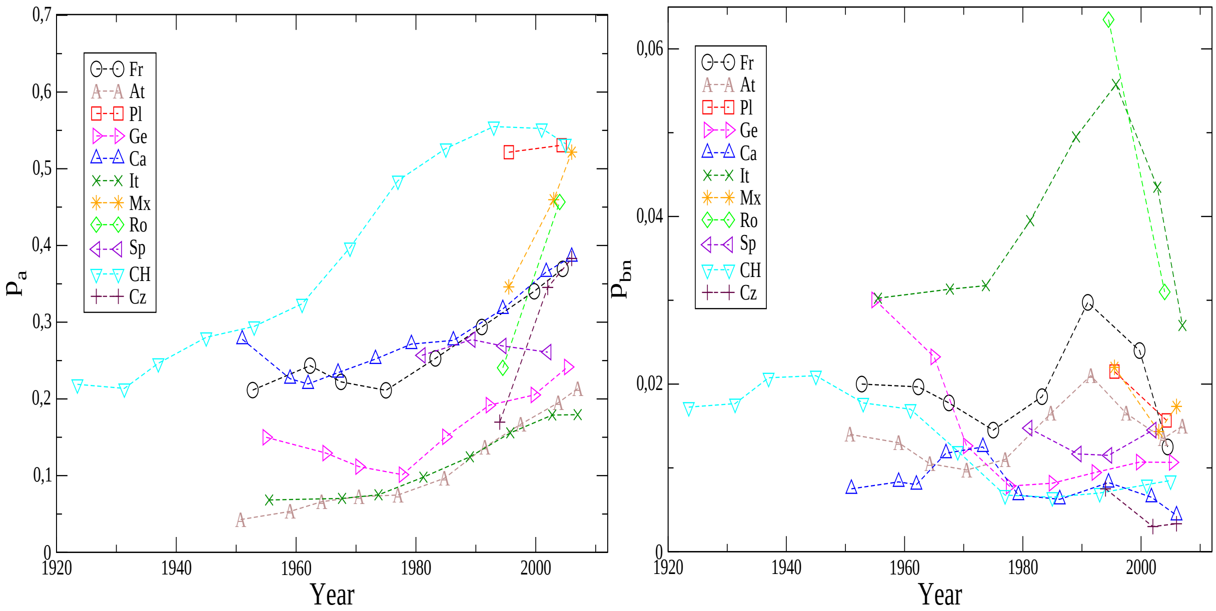

Figure S2: **Moving average, as a function of time, per country of p_a and p_{bn} at national scale** for Chamber of Deputies elections. The average is made over 4 elections. Left: about ratio of registered voters who do not take part to the election (p_a); Right: about Blank and Null ratio (p_{bn}).
