# Supplementary material for: Between Order and Disorder: A ‘Weak Law’ on Recent Electoral Behavior among Urban Voters?
Source: PLoS One. 2012 Jul 25;7(7):e39916. doi: 10.1371/journal.pone.0039916 (PMC3405122; doi:10.1371/journal.pone.0039916)
Supplement: Figure S3 — Histograms of for the 200 and 50 most populated municipalities, similarly to Fig. 8 -d (with 100 most populated municipalities for the latter one). (PDF) [file pone.0039916.s003.pdf]

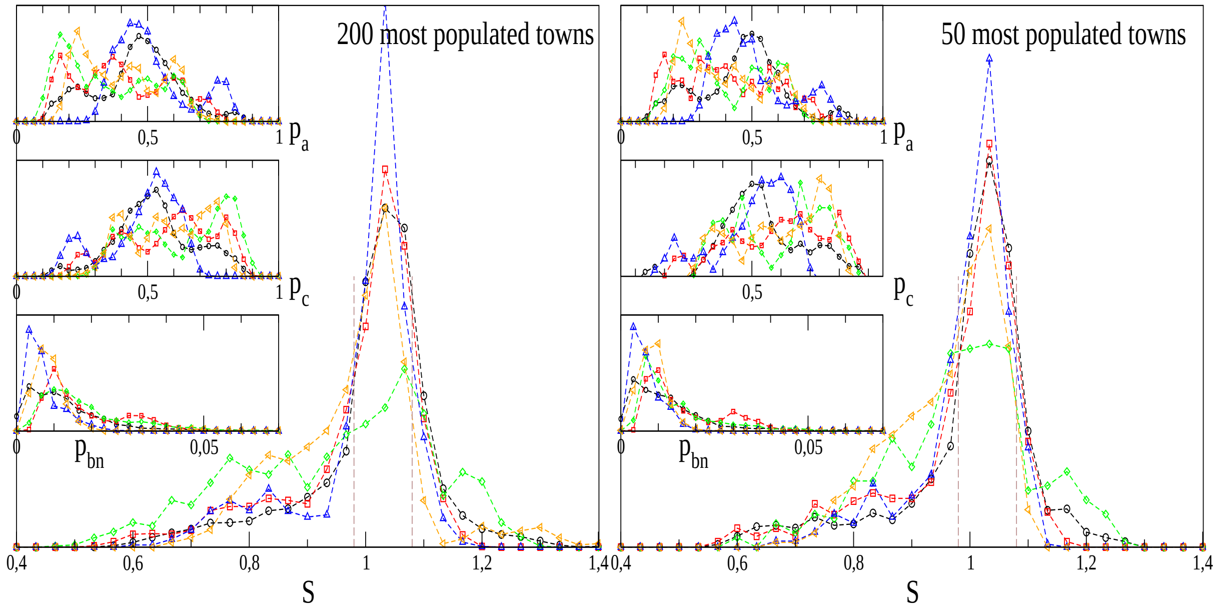

Figure S3: **Histograms of S for the $\approx$ 200 (left) and 50 (right) most populated municipalities**, similarly to Fig. 8-d (with 100 most populated municipalities for the latter one).
