# Supplementary material for: Between Order and Disorder: A ‘Weak Law’ on Recent Electoral Behavior among Urban Voters?
Source: PLoS One. 2012 Jul 25;7(7):e39916. doi: 10.1371/journal.pone.0039916 (PMC3405122; doi:10.1371/journal.pone.0039916)
Supplement: Figure S4 — Evolution in time of scatter plots of at national level of 321 elections. (PDF) [file pone.0039916.s004.pdf]

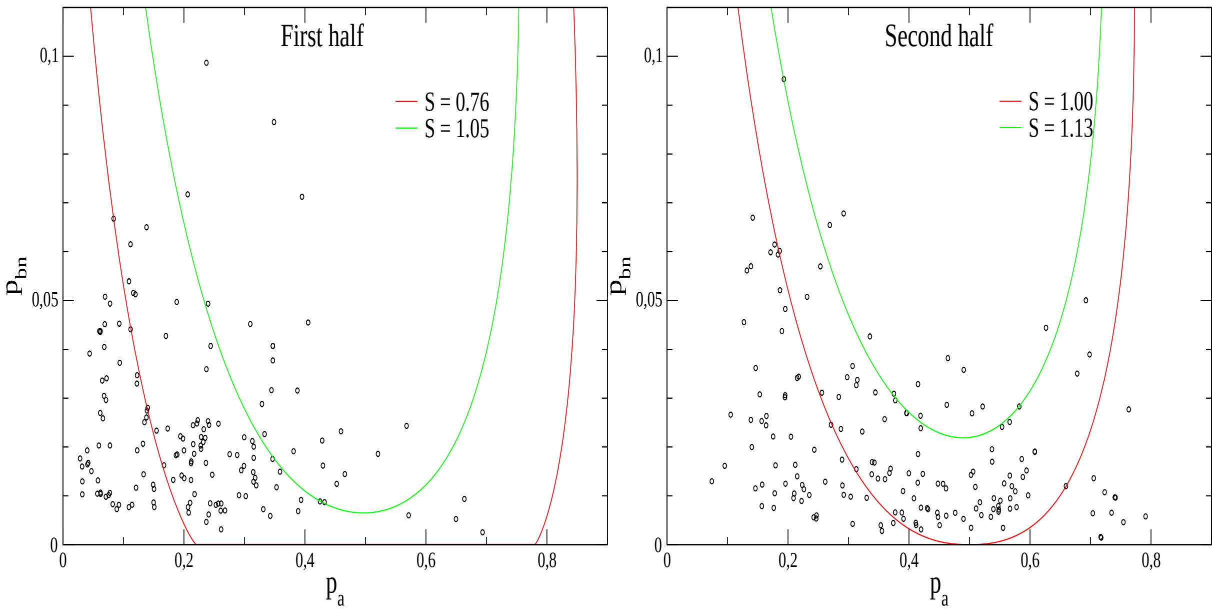

Figure S4: **Evolution in time of scatter plots of** $(p_a,\, p_{bn})$ **at national level** of 321 elections. Elections are divided into the two groups in the same manner as in Fig. 6. Curves give the sets of points $(p_a,\, p_{bn})$ such that $S(p_a, p_{bn})$ is equal to one of the two endpoints of the minimal interval of S which contains 50% of events. Note if S is equal to the average value (weighted by the population size) at lower aggregate scale (as provinces, *départements*, etc.) like in Fig. 6, the peak of S near $S \approx 1$ would be more narrowed and more centered on $S = 1$
