# Supplementary material for: Between Order and Disorder: A ‘Weak Law’ on Recent Electoral Behavior among Urban Voters?
Source: PLoS One. 2012 Jul 25;7(7):e39916. doi: 10.1371/journal.pone.0039916 (PMC3405122; doi:10.1371/journal.pone.0039916)
Supplement: Figure S5 — Scatter plots of of French municipalities according to their relative population size, over elections since 2000 (similarly as in Fig. 8-b, c, d) . (PDF) [file pone.0039916.s005.pdf]

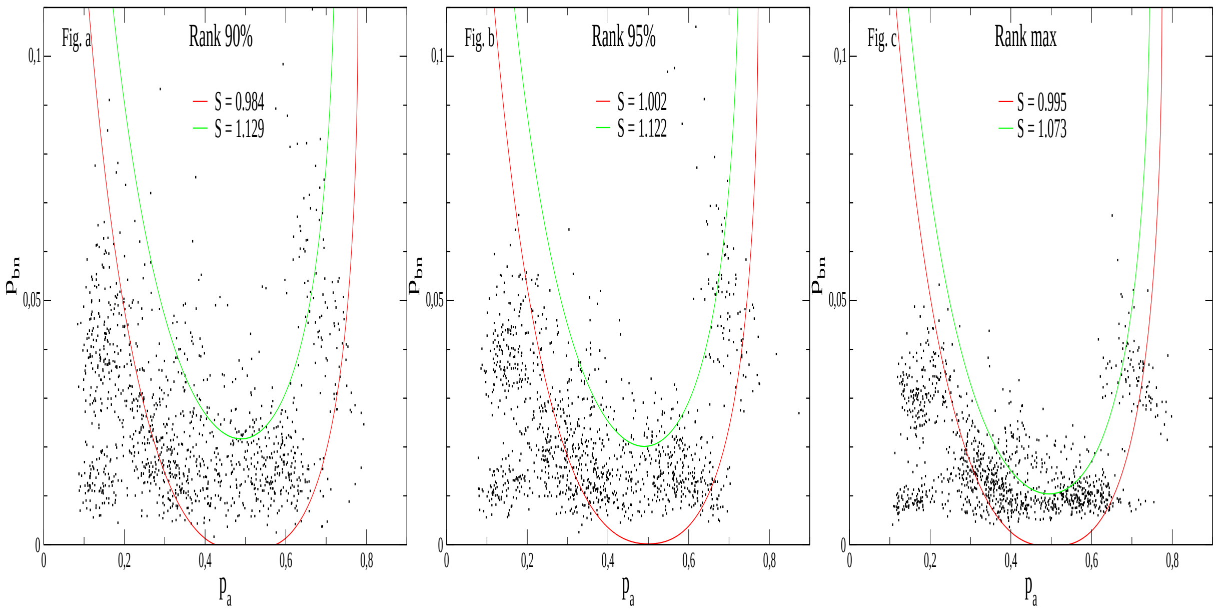

Figure S5: **Scatter plots of (p_a, p_{bn}) of French municipalities according to their relative population size**, over elections since 2000 (similarly as in Fig. 8-b, c, d). The sets of points (p_a, p_{bn}) such that $S(p_a, p_{bn})$ is equal to one of the two endpoints of the minimal interval of S which contains 50% of events (as in Fig. 9 for the most populated municipalities) are also plotted.
