# Supplementary material for: Between Order and Disorder: A ‘Weak Law’ on Recent Electoral Behavior among Urban Voters?
Source: PLoS One. 2012 Jul 25;7(7):e39916. doi: 10.1371/journal.pone.0039916 (PMC3405122; doi:10.1371/journal.pone.0039916)
Supplement: Figure S6 — Histograms of of the 100 most populated towns compared with 100 artificial towns, in France, over elections since 2000. See Appendix S1, Section C, for more explanation. (PDF) [file pone.0039916.s006.pdf]

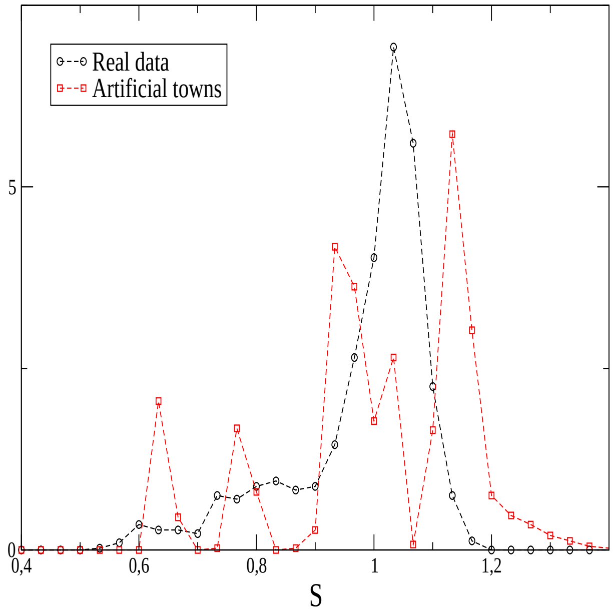

Figure S6: **Histograms of S of the 100 most populated towns compared with 100 artificial towns**, in France, over elections since 2000. See Appendix S1, Section C, for more explanation.
