# Supplementary material for: Between Order and Disorder: A ‘Weak Law’ on Recent Electoral Behavior among Urban Voters?
Source: PLoS One. 2012 Jul 25;7(7):e39916. doi: 10.1371/journal.pone.0039916 (PMC3405122; doi:10.1371/journal.pone.0039916)
Supplement: Appendix S1 — A. Details on the data sources; B. More details on data analysis; C. Finite size effects; D. Logarithmic three choices value, , of polling stations; E. Looking for signs of tension, through polling stations analysis; F. Disentangling Blank votes from Null votes (PDF) [file pone.0039916.s007.pdf]

Between order and disorder: a 'weak law' on recent electoral behavior among urban voters?

Christian Borghesi, Jean Chiche and Jean-Pierre Nadal

Supporting Information: Appendix S1

A. Data

• Elections studied at municipality scale

Table S1 gives more details about the 76 elections studied in this paper at the municipality scale. There are: 13 elections from Austria [1] ($\approx$ 2400 municipalities) [1]; 5 from Canada [2] ($\approx$ 7700 municipalities); 1 from Czech Republic [3] ($\approx$ 6400 municipalities); 20 from Metropolitan France [4] ($\approx$ 36000 municipalities); 7 from Germany [5] ($\approx$ 12000 municipalities) [2]; 4 from Italy [6] ($\approx$ 8100 municipalities); 4 from Mexico [7] ($\approx$ 2400 municipalities) [3]; 11 from Poland [8] ($\approx$ 2500 municipalities) [4]; 4 from Romania [9] ($\approx$ 3200 municipalities) [5] [6], 4 from Spain [10] ($\approx$ 8100 municipalities) and 3 from Switzerland [11] ($\approx$ 2700 municipalities) [7].

Table S1 also gives basic statistics over the $\approx$ 100 ($\approx$ 200 for France) most populated municipalities.

• Time evolution at the national or provincial scale

The study of time evolution of S is done for the same countries as in Tab. S1 and for all national elections for which we have enough data. For Austria [12], the study considers data since 1945, even if compulsory voting was abolished in the whole country in 1992 for National Council elections (D), and after 2004 for Presidential elections (P) (but in 1982 some provinces had yet done it); for Canada [13], since 1945; for Czech Republic [14], since 1990 [8]; for France [15], since 1945 [9]; for Germany [16], since 1949; for Italy [17], since 1945 even if there were compulsory voting until 1993 [10]; for Mexico [18], since 1991; for Poland [19],

[1] Corrections due to *wahlkarten* or postal votes are taking account from the national level, i.e. in this paper, each municipality receive from voting cards a number of votes and valid votes proportional to its number of population, and at the same ratio for every municipality.

[2] Chamber of Deputies (D) elections refer to the *German Bundestag* elections. *Land* Parliament elections at time less or equal to 2004 (or 2010) in each *Land* are written here as '2004 Ld' (or '2010 Ld'). Postal votes (*briehwahlen*) are usually taken account at *Landkreis* scale (they are distributed in municipalities, according to their populations), when it is it possible to do it. Nevertheless, these corrections provide a very small difference in Fig. 5, especially for high population-size bins.

[3] The 2006 *Senador* election (not studied here) gives a very near statistics of S than the (P) and (D) elections that also occur at the same time.

[4] The Chamber of Deputies (D) election is the *Sejm* Chamber election.

[5] The referendum studied here is about *the reduction of the number of parliamentarians to a number of 300 persons*, and not about the *adoption of a unicameral Parliament* held on the same time. The latter one is not known at the polling station level.

[6] Some Romanian electors, not registered in the *lista electorala permanenta*, are able to vote. For this country, we pursue to write N the Number of Register Voters, N_v the registered electors who take part to the election, and N_{bn} the number of Null and Blank Votes that the Registered Voters could make (even if the latter data is not known.) Romanian electoral data gather for each municipality, N, N_v, $N_v(tot)$ (the total number of votes), and $N_{bn}(tot)$ the total number of Null and Blank votes. Assuming that registered electors and not registered electors vote Null and Blank in the same way (i.e. $\frac{N_{bn}(tot)}{N_v(tot)} = \frac{N_{bn}}{N_v}$), we deduce N_{bn}.

[7] The referendums or *votations* 'R(a)' and 'R(b)' respectively occurred the 11 of March and the 17 of June. The Legislative (D) election refers to the *Conseil National* election.

[8] The 1990 and 1992 Deputies (D) elections only refer to the Parliamentary Chamber of People election. The Parliamentary Chamber of Nations and the Parliamentary National Council elections, that occurred at the same day as the previous ones, also gave approximately the same S value.

[9] All French electoral data are from metropolitan France. Some referendums are not known at the *département* scale. In these cases, S is evaluated at the national scale.

[10] We consider the only first question asked to electors in referendums.

Id	$\overline{S}$	$\overline{p_a}$	$\overline{p_c}$	$\overline{p_{bn}}(\overline{p_b})$	Id	$\overline{S}$	$\overline{p_a}$	$\overline{p_c}$	$\overline{p_{bn}}(\overline{p_b})$
Fr 1992 R	**1.02**±0.04	0.32	0.66	0.018	Fr 1993 D	1.09±0.04	0.34	0.63	0.028
Fr 1994 E	1.12±0.03	0.48	0.50	0.020	Fr 1995 P1	0.91±0.04	0.24	0.74	0.018
Fr 1995 P2	**1.01**±0.07	0.23	0.73	0.044	Fr 1997 D	**1.08**±0.03	0.36	0.62	0.024
Fr 1998 rg	1.11±0.03	0.46	0.52	0.019	Fr 1999 E	1.11±0.03	0.54	0.44	0.020
Fr 2000 R	**1.02**±0.07	0.71	0.25	0.036	Fr 2002 P1	**1.01**±0.04	0.31	0.67	0.019
Fr 2002 P2	0.95±0.07	0.21	0.75	0.035	Fr 2002 D	**1.02**±0.04	0.37	0.62	0.010
Fr 2004 rg	1.10±0.04	0.41	0.57	0.021	Fr 2004 E	**1.04**±0.03	0.57	0.42	0.010
Fr 2005 R	**1.00**±0.05	0.32	0.66	0.014	Fr 2007 P1	0.72±0.08	0.17	0.82	0.010
Fr 2007 P2	0.84±0.06	0.17	0.80	0.032	Fr 2007 D	**1.04**±0.03	0.42	0.57	0.009
Fr 2009 E	**1.03**±0.05	0.60	0.39	0.012	Fr 2010 rg	**1.06**±0.03	0.57	0.42	0.012
At 1994 D	0.81±0.11	0.20	0.78	0.016	At 1995 D	0.73±0.10	0.15	0.83	0.018
At 1996 E	**1.04**±0.04	0.33	0.65	0.021	At 1998 P	**1.00**±0.10	0.27	0.70	0.032
At 1999 E	**1.06**±0.05	0.52	0.46	0.013	At 1999 D	0.82±0.09	0.22	0.77	0.011
At 2002 D	0.73±0.10	0.17	0.81	0.011	At 2004 P	**1.04**±0.09	0.31	0.66	0.028
At 2004 E	**1.03**±0.05	0.59	0.40	0.010	At 2006 D	0.87±0.09	0.24	0.74	0.012
At 2008 D	0.88±0.08	0.24	0.75	0.014	At 2009 E	**1.04**±0.04	0.55	0.44	0.009
At 2010 P	1.16±0.06	0.48	0.49	0.034					
Pl 2000 P1	**0.98**±0.03	0.36	0.63	0.006	Pl 2001 D	1.09±0.02	0.52	0.46	0.015
Pl 2003 R	**0.98**±0.02	0.37	0.62	0.004	Pl 2004 E	0.79±0.07	0.78	0.22	0.005
Pl 2005 D	**1.06**±0.03	0.58	0.41	0.013	Pl 2005 P1	**1.02**±0.01	0.49	0.51	0.003
Pl 2005 P2	**1.03**±0.01	0.47	0.53	0.006	Pl 2007 D	**1.05**±0.03	0.42	0.57	0.010
Pl 2009 E	0.87±0.06	0.73	0.27	0.004	Pl 2010 P1	**1.01**±0.02	0.43	0.57	0.004
Pl 2010 P2	**1.03**±0.02	0.43	0.56	0.007					
Ge 2002 D	0.83±0.07	0.22	0.77	0.009	Ge 2004 Ld	**1.02**±0.04	0.41	0.58	0.007
Ge 2004 E	**1.02**±0.05	0.59	0.40	0.009	Ge 2005 D	0.87±0.06	0.24	0.75	0.011
Ge 2009 E	**1.00**±0.05	0.60	0.40	0.006	Ge 2009 D	0.95±0.05	0.30	0.69	0.009
Ge 2010 Ld	**1.04**±0.03	0.43	0.56	0.009					
Ca 1997 D	**1.00**±0.04	0.37	0.62	0.009	Ca 2000 D	**1.03**±0.03	0.44	0.56	0.006
Ca 2004 D	**1.02**±0.02	0.46	0.54	0.004	Ca 2006 D	**1.01**±0.02	0.44	0.56	0.003
Ca 2008 D	**1.02**±0.02	0.49	0.51	0.003					
It 2004 E	1.11±0.12	0.29	0.66	0.053(0.023)	It 2006 D	0.78±0.13	0.17	0.81	0.020(0.007)
It 2008 D	0.89±0.12	0.20	0.77	0.027(0.008)	It 2009 E	1.08±0.10	0.36	0.61	0.034(0.013)
Mx 2003 D	**1.04**±0.05	0.59	0.40	0.013	Mx 2006 D	**1.04**±0.04	0.40	0.58	0.012
Mx 2006 P	**1.03**±0.04	0.40	0.59	0.010	Mx 2009 D	1.11±0.06	0.56	0.41	0.027
Ro 2009 E	0.73±0.09	0.81	0.18	0.008	Ro 2009 R	1.09±0.02	0.55	0.44	0.017
Ro 2009 P1	**1.05**±0.02	0.52	0.48	0.008	Ro 2009 P2	**1.04**±0.02	0.50	0.50	0.006
Sp 2004 D	0.92±0.07	0.24	0.74	0.020(0.014)	Sp 2004 E	**1.01**±0.06	0.57	0.42	0.006(0.003)
Sp 2008 D	0.91±0.08	0.26	0.73	0.013(0.009)	Sp 2009 E	**1.03**±0.04	0.56	0.43	0.009(0.006)
CH 2007 R(a)	**1.04**±0.04	0.53	0.46	0.008(0.004)	CH 2007 R(b)	**0.99**±0.06	0.62	0.37	0.007(0.004)
CH 2007 D	**1.04**±0.05	0.53	0.47	0.009(0.002)					
Cz 2003 R	**1.07**±0.01	0.47	0.52	0.012					

Table S1: **Elections studied in this paper at the municipality scale.** An election is identified (Id) by its country, its year date and its nature. D: Chamber of Deputies election; E: European parliament election; P: presidential election (according to the constitution of the country, in only one round); P1 and P2: first and second round of a Presidential election; R: Referendum; Ld: German *Länder* elections; rg: French *Régionales* elections. For each country elections are given in a chronological order (but the 2006 Mexican Presidential (P) and Deputies (D) elections occurred the same day, and also for the 2009 Romanian Presidential (P1) and Referendum (R) elections). Even if an election needs two rounds, only the first one is considered (e.g. the French Deputies (D) and *Régionales* (rg) elections) unless the contrary is indicated (e.g. P1 and P2). Mean values of S, p_a, p_c, p_{bn}(and (p_b) if Blank Vote are distinguished between Null Vote), and also standard deviation only for S, are given over the bin of the ≈ 100 (or ≈ 200 for France only) most populated municipalities. In bold text, $\overline{S} \in [0.98; 1.08]$.

Country	Kind of elections	Scale of aggregate data
At	D, E, P, R	National
Ca	D	Province (5-13)
CH	D, R	*Canton* (25-26)
Cz	D, E, R, rg, S1, S2	National
Fr	Cant, D, E, P1, P2, R, rg	*département* (90-96)
Ge	D, E	*Land* (9-16)
It	D, E, R, S	National
Mx	D, P	National
Pl	D, E, P1, P2	National
Ro	D, E, P1, P2, R	National
Sp	D, E, R	*Comunidad autónoma* (17-19)

Table S2: **Elections studied in this paper at large scale for their evolution in time.** Notation is the same as in Tab. S1. For Czech Republic, "rg" means Election into regional councils, "S1" and "S2" are respectively the first and second round of the Senate elections; for France, "Cant" refers to the *Cantonales* elections and some referendums are only known at the national scale; for Italy, "S" means Senate elections, and occur at the same time as Deputies elections (D) but with older registered voters. In parenthesis, the total number of different provinces (or *Cantons*, etc.), which can change in time, in the whole country.

since 1990 [11]; for Romania [20], since 1990; for Spain [21], since 1976; for Switzerland [22], since 1884 for referendums (R) and since 1919 for legislative elections (D). If an election needs two rounds, the first one is considered, unless the contrary is indicated. The Mexican, Polish and Romanian Senate elections are not shown here because they occur at the same time as Chamber of Deputies elections and have very similar S results.

Table S2 summarizes the nature of elections studied in this paper, and also the scale of aggregate data per country. Note that the last election analyzed in this paper is the Referendum which held in Italy on June 2011. [12]

Websites given in the References were accessed in December 2011. Part of the database used in this paper can also be directly downloaded from [23].

• Elections studied at polling station scale

Polling stations analysis is restricted to polling stations which belong to one of the 100 most populated municipalities (for the considered election). 31 elections at the polling station scale are studied in this paper: 5 for Canada (each Canadian election of Tab. S1), with around 25000 polling stations; 13 for France (French elections of Tab. S1 since 1999), with around 7000 polling stations; 4 for Mexico (each Mexican election of Tab. S1), with around 55000 polling stations or ballot box; 5 for Poland (Polish election of Tab. S1 from 2003 up to 2005), with around 8000 polling stations; and 4 for Romania (each Romanian election of Tab. S1), with around 6000 polling stations. See Tab. S3 for some basic statistics over polling stations of the 100 most populated municipalities.

[11] We have not data from the 1989 Chamber of Deputies (Sejm) election nor the two referendums in 1996.

[12] Official results (which took into account registered voters) of the Canadian Chamber of Deputies election, held on May 2011, were not published at the time we first submitted this paper. In Fig. S1, the involvement entropy over all provinces would be $\approx 1.00 \pm 0.02$ and respectively 0.99 and 1.02 for Ontario and Quebec.

Id	S	τ_3	Id	S	τ_3
Fr 1999 E	1.09 ± 0.05	-3.7 ± 0.6	Fr 2000 R	1.00 ± 0.11	-4.2 ± 0.6
Fr 2002 P1	1.00 ± 0.06	-2.1 ± 0.6	Fr 2002 P2	0.93 ± 0.10	-0.7 ± 0.6
Fr 2002 D	1.01 ± 0.06	-3.2 ± 0.7	Fr 2004 rg	1.09 ± 0.05	-2.8 ± 0.6
Fr 2004 E	1.03 ± 0.05	-4.6 ± 0.7	Fr 2005 R	0.99 ± 0.07	-2.5 ± 0.7
Fr 2007 P1	0.71 ± 0.11	-1.3 ± 0.7	Fr 2007 P2	0.83 ± 0.09	-0.1 ± 0.7
Fr 2007 D	1.03 ± 0.04	-3.8 ± 0.7	Fr 2009 E	1.02 ± 0.07	-4.6 ± 0.7
Fr 2010 rg	1.04 ± 0.05	-4.4 ± 0.7			
Ca 1997 D	0.98 ± 0.08	-3.3 ± 1.3	Ca 2000 D	1.00 ± 0.06	-4.1 ± 1.1
Ca 2004 D	1.00 ± 0.05	-4.4 ± 0.9	Ca 2006 D	0.99 ± 0.05	-4.4 ± 0.8
Ca 2008 D	1.00 ± 0.05	-4.6 ± 0.9			
Pl 2003 R	0.95 ± 0.10	-4.0 ± 0.9	Pl 2004 E	0.83 ± 0.13	-6.3 ± 0.8
Pl 2005 D	1.05 ± 0.08	-4.1 ± 0.9	Pl 2005 P1	1.00 ± 0.07	-4.9 ± 0.9
Pl 2005 P2	1.01 ± 0.05	-4.1 ± 0.9			
Mx 2003 D	1.03 ± 0.07	-4.3 ± 0.9	Mx 2006 D	1.02 ± 0.07	-3.2 ± 0.8
Mx 2006 P	1.01 ± 0.07	-3.4 ± 0.8	Mx 2009 D	1.11 ± 0.10	-3.5 ± 0.9
Ro 2009 E	0.70 ± 0.13	-6.6 ± 0.9	Ro 2009 R	1.08 ± 0.05	-3.8 ± 0.7
Ro 2009 P1	1.04 ± 0.03	-4.5 ± 0.7	Ro 2009 P2	1.04 ± 0.03	-4.4 ± 0.7

Table S3: **Elections studied at the polling station level.** An election is identified (Id) by its country, its year date and its nature. Mean value and standard deviation of S and of τ_3 (see the SI Section D) over ballot boxes in the 100 most populated municipalities.

B. More details on data analysis

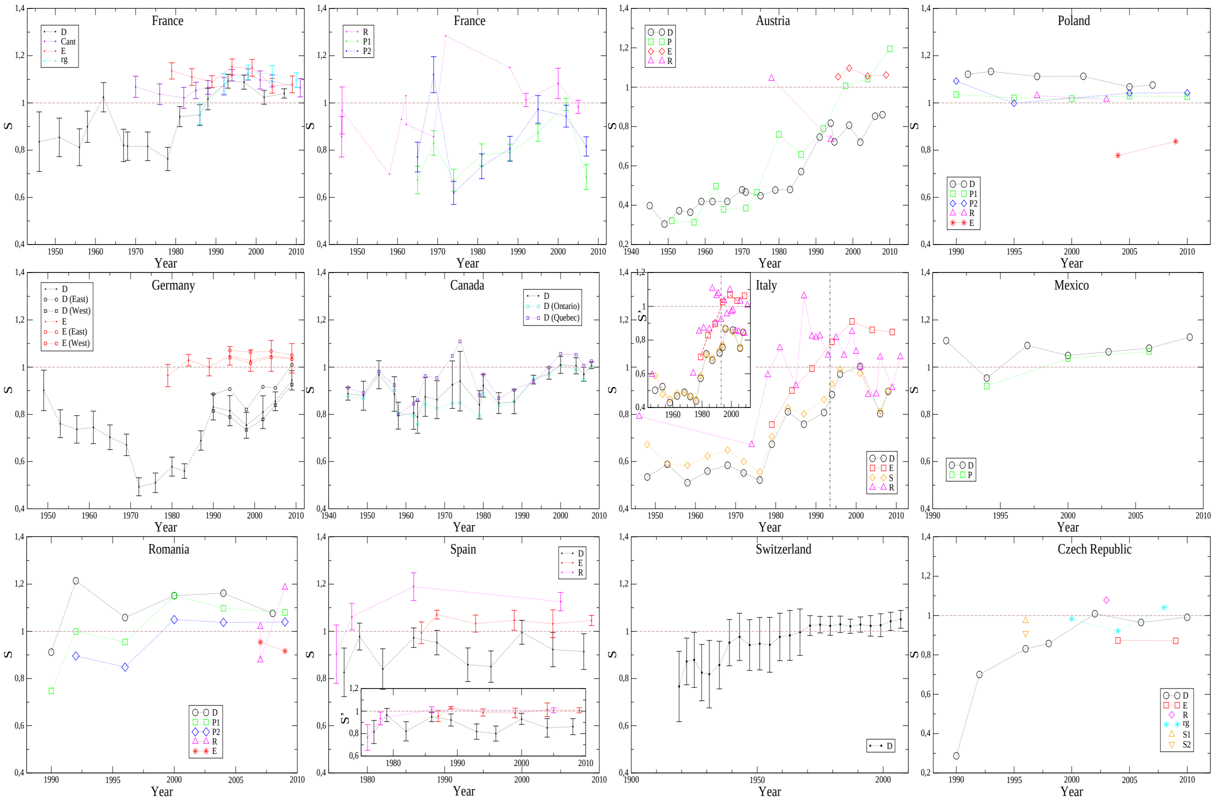

Figure S1: **Time evolution of the mean involvement entropy at large scale** (national, provincial, etc.). See Section A and Tab. S2, for more details and also for the end of compulsory voting in Italy (cf. vertical dashed line) and in Austria. Whenever the scale of aggregate data is lower than the national one, standard-deviations (weighted by the number of registered voters) are also shown as error bars. Italian and Spanish graph insets show a variant of S where Blank Votes are categorized as Valid Votes (see Section F for more discussion). See text for more explanation about some French curves.

Fig. S1 gathers all the available data (see in the SI, Section A for more details) at a large aggregate scale (country, province, *département*, etc.). When the scale of aggregate data is lower than the national one, each point corresponds to a weighted (by population-size) mean value of involvement entropies at lower scale (province, *département*, etc.), and standard deviation is also given as error bar. The cases where Blank Votes are distinguished from Null Votes (i.e. in Italy, Spain and Switzerland), call for a specific discussion (see the SI, Section F).

Let us comment Fig. S1 on the case of the Chamber of Deputies elections in France, at the large scale called *département* (96 in quantity for metropolitan France, actually). One sees an involvement entropy frequently equal to ≈ 0.8 until 1981, which then increases and gets greater than 1 until 2000, and decreases a little and stabilizes to $S \approx 1$after 2000. So, the civic involvement of the electorate (at the *département* scale) is relatively ordered until 1981 and get more and more disordered until 2000. After 2000, S seems to stabilize to a common value $S \approx 1$ which is also reached for the European Parliament elections and for local elections at different scales, such as the *Régionales* ($\sim$ states) and the *Cantonales* ($\sim$ counties) elections.

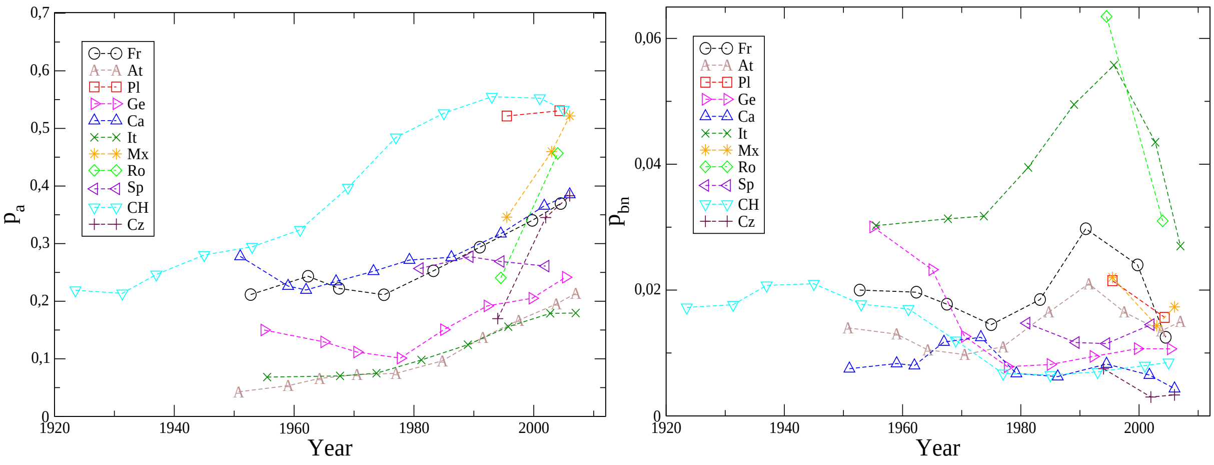

Figure S2: **Moving average, as a function of time, per country of p_a and p_{bn} at national scale** for Chamber of Deputies elections. The average is made over 4 elections. Left: about ratio of registered voters who do not take part to the election (p_a); Right: about Blank and Null ratio (p_{bn}).

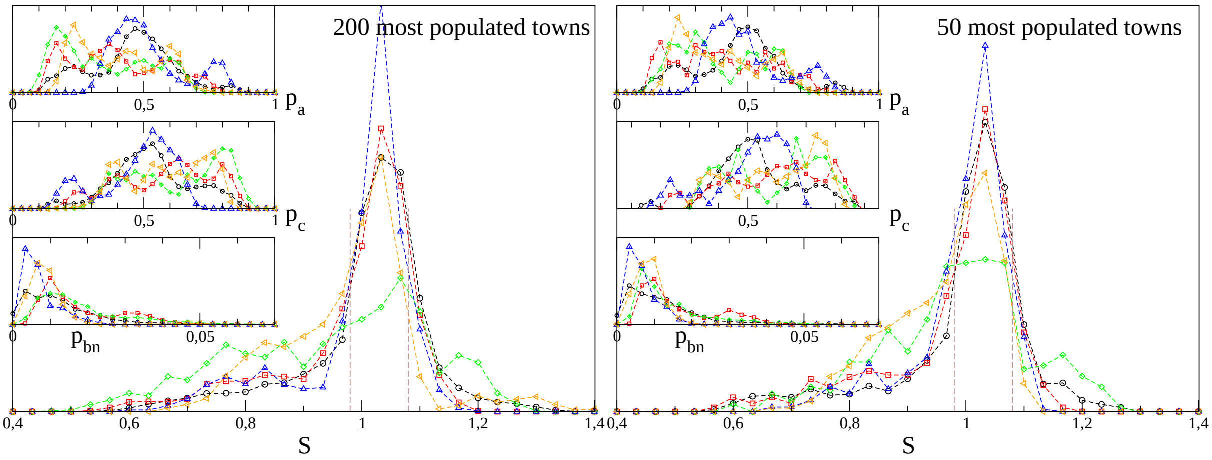

Figure S3: **Histograms of S for the ≈ 200 (left) and 50 (right) most populated municipalities**, similarly to Fig. 8-d (with 100 most populated municipalities for the latter one).

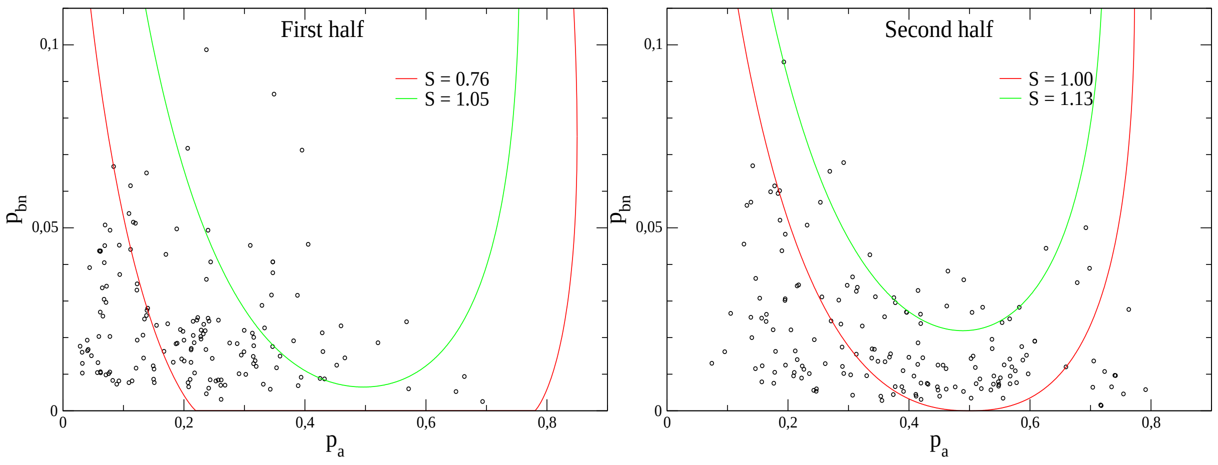

Figure S4: **Evolution in time of scatter plots of** $(p_a,\, p_{bn})$ **at national level** of 321 elections. Elections are divided into the two groups in the same manner as in Fig. 6. Curves give the sets of points $(p_a,\, p_{bn})$ such that $S(p_a, p_{bn})$ is equal to one of the two endpoints of the minimal interval of S which contains 50% of events. Note if S is equal to the average value (weighted by the population size) at lower aggregate scale (as provinces, *départements*, etc.) like in Fig. 6, the peak of S near $S \approx 1$ would be more narrowed and more centered on $S = 1$

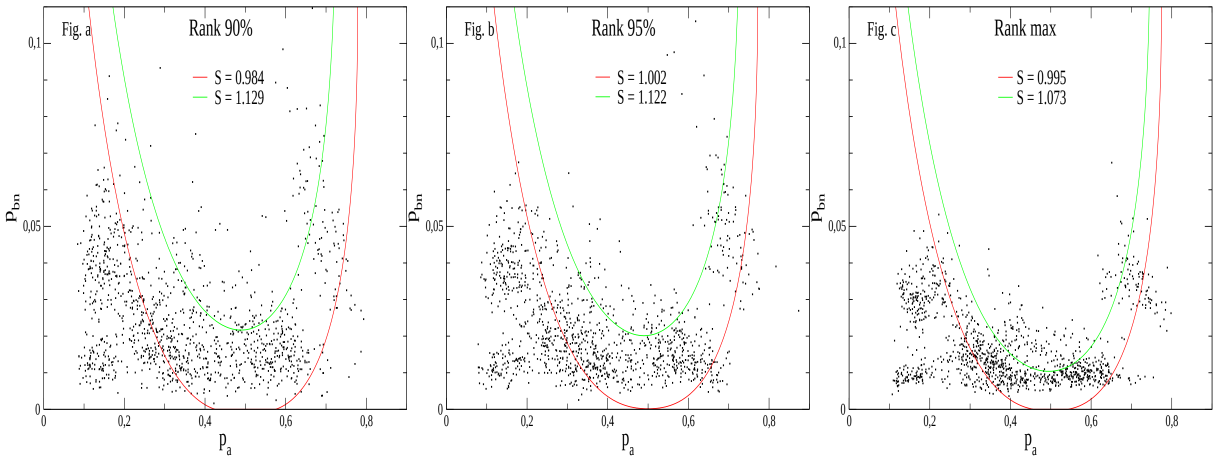

Figure S5: **Scatter plots of** $(p_a,\, p_{bn})$ **of French municipalities according to their relative population size**, over elections since 2000 (similarly as in Fig. 8-b, c, d). The sets of points $(p_a,\, p_{bn})$ such that $S(p_a, p_{bn})$ is equal to one of the two endpoints of the minimal interval of S which contains 50% of events (as in Fig. 9 for the most populated municipalities) are also plotted.

C. Finite size effects

We show in this section that finite size effects over municipality-size, N, on the entropy-involvement S, are relatively small for the most populated municipalities. Biases due to finite size effects have two possible origins: (1) level of aggregation of the data, over N about a hundred to a million, influences S measures, and (2) a statistical effect due to large numbers. Without a loss of generality, we examine these two biases for French electoral data – with 20 elections at the municipality scale and 13 at the polling station level, cf. the SI Section A. Lastly we show that the distribution of the involvement entropy which is sharply peaked near $S \approx 1$ for most populated towns is not due to considering a large number of N per town.

(1) Scale at which data are aggregated

French municipality sizes range from around 10 to around $100,000$. In order to investigate how aggregate data scale modifies the measurement of the involvement entropy S, for each municipality we compare the results at the municipality scale with the one done at the polling station scale. Registered voters per polling station do not exceed around one thousand in France. We compare for a municipality its involvement entropy, S, measured at the municipality level, to the mean value, S_{PS}, of the involvement entropy over all the polling stations in the the considered municipality. Convexity of the logarithmic function implies that the later is at most equal to the former. For each of the 200 most populated French municipalities, and for each of the 13 French elections known at the polling station scale (see the SI Section A), the gap between S and S_{PS} is less than about 2% (except for very few and typical recording errors of electoral data). Moreover, averaging S and S_{PS} over samples of ≈ 200 municipalities of similar sizes N provides a difference less than 1% for $N \gtrsim 1000$.

In short, for large population municipalities, the bias introduced by the scale at which data are aggregated is weak and does not affect the main conclusions of the paper.

(2) Statistical effects due to large numbers

Let us see if statistical fluctuations due to finite size effects considerably modify the expected values of involvement entropy. Indeed, For independent events, according to the central limit theorem (under conditions broadly applicable) fluctuations are on the order of $1/\sqrt{N}$. This is expected to be the case for the ratios p_a and p_{bn}, which should then lead to a bias in the entropy value. We want to estimate this bias and see if it is negligible (say less than 1%). To do so, we make a simulation with artificial data. For calibrating these data, we make use of the sample of the most populated municipalities. We measure the average values $\overline{p_a}$ and $\overline{p_{bn}}$ of p_a and p_{bn} over all municipalities in this sample of the largest municipality-size; and the corresponding standard deviations σ_a and σ_{bn}. The surrogate data consists in a same number of "municipalities", each one characterized by the same population size as in the empirical data. For these surrogate-municipalities, we draw the numbers of Abstentionists and of Null-Blank votes from binomial distributions, parametrized by the empirical average values and standard deviations of p_a and p_{bn}, as follows.

Let a surrogate-municipality with N registered voters. Its numbers of Abstentionists, N_a, and Null-Blank votes, N_{bn}, are drawn from a binomial distribution such that:

$$\begin{aligned} N_a &= \mathcal{B}(N\ ;\ \overline{p_a} + \eta_a), \\ N_{bn} &= \mathcal{B}(N\ ;\ \overline{p_{bn}} + \eta_{bn}), \end{aligned} \tag{S1}$$

where η_a and η_{bn} are independent random Gaussian noises of mean 0 and of standard deviation σ_a and σ_{bn}, respectively. Note that here, for each citizen in a surrogate-municipality, probabilities to not vote and to put a null-blank vote are mutually independent.

Now, we can compare the average values $\overline{S}(N)$ of municipal involvement entropy in a sample of $\approx N$ surrogate-municipality-size, with $\overline{S}(N_{max})$ in the sample of the most populated municipalities. We find

that the difference is less than 1% when $N \gtrsim 2000$. In other words, for municipality-size greater than around 2000, statistical fluctuations due to finite size effects are negligible for what concern the present study.

To conclude, we have seen that, for French electoral data, finite size effects do not affect significantly the municipal involvement entropy (i.e. by less than a 2% deviation) for N greater than 2000. Note that 2000 is much less than the typical municipality size of the most populated municipalities, for which the common value $S \approx 1$ is frequently found. Lastly, the same analysis done for other countries for which electoral data are also available at the polling station scale (see the SI Section A) give the same results (see e.g. mean values of S over the 100 most populated municipalities, at the municipality scale in Tab. S1, compared to those at ballot box scale in Tab. S3).

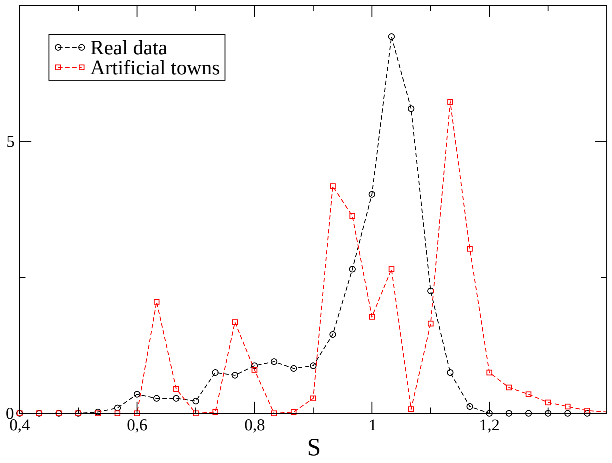

Figure S6: **Histograms of S of the 100 most populated towns compared with 100 artificial towns** (see text), in France, over elections since 2000.

Now, let us show that the shape of the distribution of S over the 100 most populated towns (which is sharply peaked near $S \approx 1$, apart from Austria) does not result from aggregating a large number of the citizen choices. In other words, the shape of the distribution of the involvement entropy for the 100 most populated towns (cf. Fig. 8-d) cannot be explained by a statistical bias due to a large number effect.

In order to see this point, 100 artificial town is created – in France, without he loss of generality. Each artificial town results from the aggregation over 300 real small municipalities of real numbers of registered voters (N), abstentionists (N_a), blank and null votes (N_{bn}) and votes according to the list of choices (N_c). In other words, an artificial town comes from the aggregation of real citizen choices who live in small municipalities. Each municipality is taken into account only once. These 100 French artificial towns have artificial aggregated registered voters (N) from 7000 to 330000, and is equal to 34000 in average. Fig. S6 allows one to compare the real distribution of S of the most populated French towns over elections since 2000 with the one which results from these 100 artificial towns. These two histograms are clearly different.

To conclude, the shape of the distribution of the involvement entropy of most populated towns (cf. Fig. 8-d) is not due to a bias rooted in aggregating a large number of citizen choices. The shape itself depends on real citizen choices who live in these towns.

D. Logarithmic three choices value, τ_3, of polling stations

As a supplement to the study of the entropy defined from the set of three ratios $\{p_a, p_c, p_{bn}\}$, in this section we introduce another variable, called logarithmic three choices value, τ_3, which also takes into account the set $\{p_a, p_c, p_{bn}\}$. First, we show that the distribution of τ_3, over polling stations in the 100 most populated municipalities appears stable over time, and also similar between different countries. Secondly, we justify our interest for this logarithmic three choices value from hypothesis on agents behavior. We compare two simple decision making rules, and give arguments against the most intuitive one, that of a choice decomposed into two successive binary choice decisions (first to vote or not, then to cast a valid vote or not). This confirms in a different way the existence of correlations between p_a and p_{bn} (see Main text, Results Section, paragraph on "Abstentions, valid votes and blank or null votes").

D.1. Logarithmic three choices value of polling stations in most populated towns

In this section we generalize the analysis done in [24, 25], where the statistics of the logarithmic turnout rate, $\tau = \ln \frac{p_a}{1-p_a}$, is studied. When considering the three possible values, p_a, p_{bn}, p_{bn}, the logarithmic three choices value τ_3, as justified below Section D.2, can be defined by

$$\tau_3 = \ln \Big(\frac{p_c \cdot p_{bn}}{(p_a)^2} \Big). \tag{S2}$$

Fig. S7 shows the pdf of the logarithmic three choices value τ_3 over different polling stations of the 100 most populated towns in each country (apart from Canadian ones because more than third of polling stations have $p_{bn} = 0$, which lead to their logarithmic three choices values τ_3 are undefined), i.e. the probability $P(\tau_3)d\tau_3$ that a given polling station, inside the 100 most populated towns, has τ_3 to within $d\tau_3$. Although the average $\langle \tau_3 \rangle$ over these polling stations varies quite substantially between elections (see Fig. S9 and Tab. S3), the shape of the distribution of $\tau_3 - \langle \tau_3 \rangle$ is quite stable across elections for each country.

Consider now the normalized τ_3 values, that is $\hat{\tau_3} = \frac{\tau_3 - \langle \tau_3 \rangle}{\sigma}$, where $\langle \tau_3 \rangle$ and σ are respectively the mean value and the standard deviation of τ_3 over polling stations of the 100 most populated municipalities. Fig. S8 shows that the remarkable similarity between the distributions of these normalized logarithmic three choices for French, Mexican, Romanian and half Polish elections. A Kolmogorov-Smirnov test, where one only allows for a relative shift of the normalized distributions $P(\hat{\tau_3})$, over polling stations of the 100 most populated towns, does not allow one to reject the hypothesis that the distribution $P(\hat{\tau_3})$ is the same for all elections (except for half of the Polish elections).

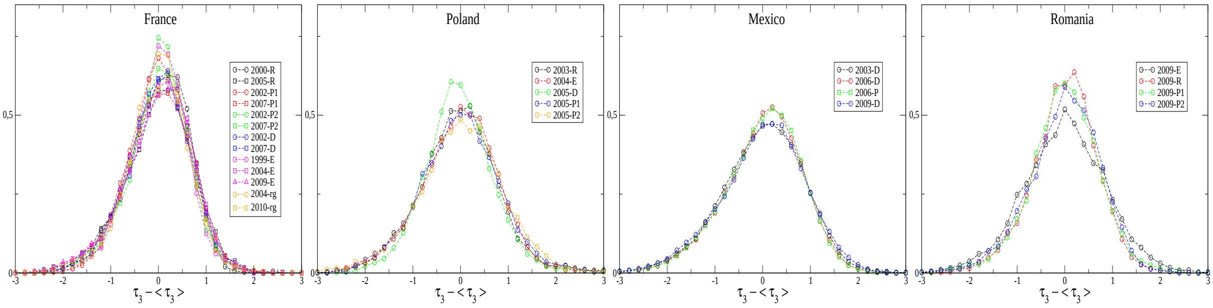

Figure S7: **Distribution over polling stations of the 100 most populated towns of $P(\tau_3 - \langle \tau \rangle)$ for each election**, where τ_3 is the logarithmic three choices value and $\langle \tau_3 \rangle$ its average value over all concerned polling stations.

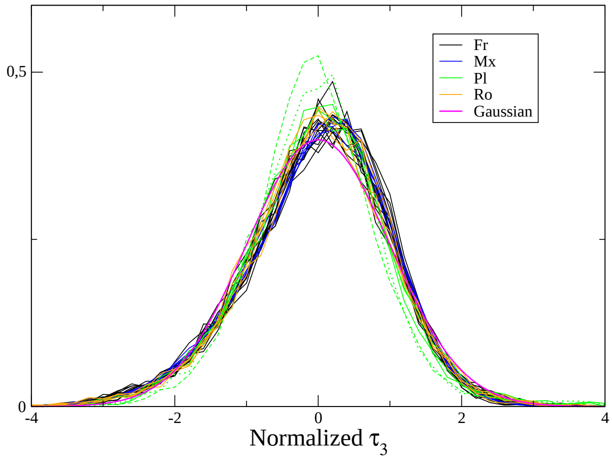

Figure S8: **Distribution of normalized τ_3 over polling stations of the 100 most populated towns** for 26 elections. The dotted line and the dashed line show respectively Pl-2003-R and Pl-2005-D elections. A normalized Gaussian is also plotted.

Remark: there is not a one-to-one relation between the logarithmic three choices value, τ_3, and the involvement entropy S. Indeed, it is enough to invoke that the three ratios $\{p_a, p_c, p_{bn}\}$ play a symmetric role for S, and not for τ_3. Fig. S9 plots τ_3 with respect to S for their average values over polling stations in each of the 100 most populated towns (see also Tab. S3 for basics statistics of S and τ_3 over polling stations in the 100 most populated municipalities).

D.2. Towards a behavioral model in the three choices case

Elaborating upon standard hypothesis on agents behavior, the goal of this section is to explain why τ_3 defined above is the natural generalization of the logarithmic turnout rate introduced in [24, 25] for a single binary choice.

- **Recall: Threshold decision rule for a single binary choice**

Let us first recall the rationale for introducing the logarithmic turnout rate. We consider N agents making a binary decision. Agent i makes its decision n_i, $n_i \in \{0, 1\}$, according to

$$n_i = \Theta(h_i + H), \tag{S3}$$

with $\Theta(x \geq 0) = 1$ and $\Theta(x < 0) = 0$. Here h_i is an idiosyncratic term characterizing the bias of agent i in favor of the decision to vote ($n_i = 1$). Idiosyncrasies are assumed independent random variables (hence uncorrelated between agents). H is a global bias, a field identically applied to all agents, which can be seen as a 'cultural field' [25]. Note that here there is no direct interaction between agents.

According to this decision rule (Eq. (S3)), in the large size limit $N \to \infty$, the fraction $p_v = 1 - p_a$ of decisions 1 among the population, is equal to the cumulative distribution of idiosyncrasies: $p_v = \mathcal{P}_>(-H) \equiv$

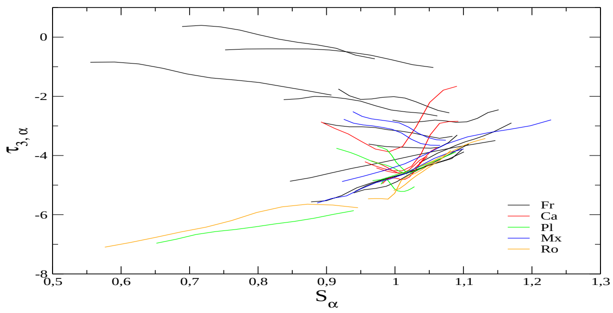

Figure S9: **Logarithmic three choices value, τ_3, with respect to involvement entropy S.** Measures come from mean values of τ_3 and S over polling stations in each of the 100 most populated towns. Curves are smoothed. Note that there is not a one-to-one relation between τ_3 and S.

$\int_{-H}^{\infty} P(h)\mathrm{d}h$. If idiosyncrasies are assumed to be distributed according to a logistic distribution [26] of zero mean and of unity width, it comes that

$$p_v = \frac{1}{1+e^{-H}}\,, \quad \text{or equivalently,}$$
$$H = \ln\big(\frac{p_v}{1-p_v}\big). \tag{S4}$$

This justifies to study the statistics of the logarithmic turnout rate $\tau = \ln\big(\frac{p_v}{1-p_v}\big)$. As shown in [25,27], the logarithmic turnout rate across French municipalities is remarkably stable over time, which allows one to make predictions that can be confronted with empirical observation [28,29].

- **Two binary decisions**

Now, we want to generalize to the case of three choices, not to vote, to cast a blank/null vote, and to cast a valid vote. One possibility is to assume a sequential decision (Figure S10, right): first to decide to vote or not, and if yes, then to decide to cast a blank/null vote, or to cast a valid vote. The alternative is to assume two mutually exclusive decisions (Figure S10, left). We explore both hypothesis, and show that data rule out the first one.

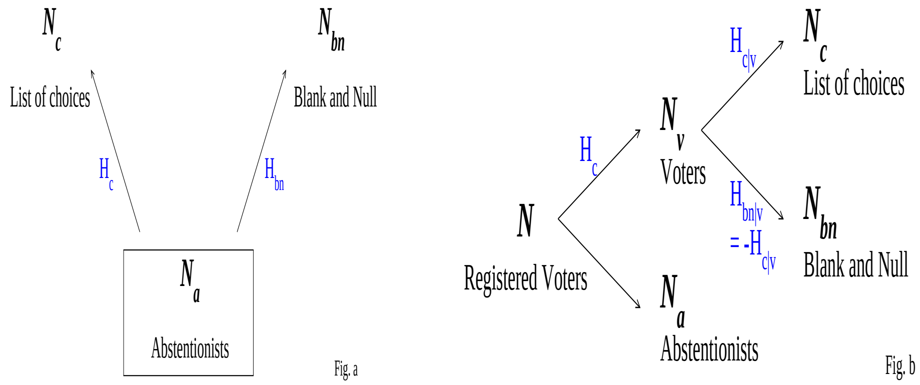

Figure S10: **Two different hypothesis on agents behavior**: (a) two mutually exclusive binary decisions; (b) two sequential binary decisions.

- **Two mutually exclusive decisions**

Here we consider that, (1), to vote according to the list of choices, and (2), to cast a blank or null vote, are two mutually exclusive decisions. In other words, abstentionists are considered like a reservoir from which agents decide to make or not the choice (1) or the choice (2); moreover if they decide to do choice (1) (or conversely (2)), they do not decide anymore to make or not the choice (2) (or conversely (1)). Let H_c the global field in favor of the choice (1) (to vote according to the list of choices), and p_c^0 the global ratio if choice (1) was unique, i.e. without any existence of choice (2) (see Fig. S10-a). Conversely, H_{bn} and p_{bn}^0 refer to choice (2) (to put a blank or null vote) if it was a unique choice. From Eq. (S4), and again assuming a logistic distribution of idiosyncrasies, it comes

$$p_c^0 = \mathcal{P}_>(-H_c), \qquad \text{thus, } H_c = \ln\big(\frac{p_c^0}{1-p_c^0}\big),$$
$$p_{bn}^0 = \mathcal{P}_>(-H_{bn}), \qquad \text{thus, } H_{bn} = \ln\big(\frac{p_{bn}^0}{1-p_{bn}^0}\big). \tag{S5}$$

Now choice (1) (respectively (2)), is made by agents who have not decided to make the other choice (2) (respectively (1)). Hence, the ratio p_c (respectively p_{bn}) is related to the ratio p_c^0 (respectively p_{bn}^0) that

would result from a single binary choice according to:

$$p_c^0 = \frac{p_c}{1-p_{bn}}, \qquad p_{bn}^0 = \frac{p_{bn}}{1-p_c}. \tag{S6}$$

Writing $H \equiv H_c + H_{bn}$, the sum of civic global fields applied to registered voters in this 3 choices process, Eqs. (S5,S6) yield to [13]

$$H = \ln\left(\frac{p_c \cdot p_{bn}}{(p_a)^2}\right). \tag{S7}$$

Hence under the hypothesis of two mutually exclusive binary decisions, the quantity to study is the logarithmic three choices rate $\tau_3 = \ln\left(\frac{p_c \cdot p_{bn}}{(p_a)^2}\right)$. This is what is done above, Section D.1.

• **Two sequential binary threshold decisions**

Now, let us consider the hypothesis of two sequential binary decisions. The first binary decision is to vote or not to vote, and the second binary decision is to decide to cast a valid vote (according to the list of choices) or to put an invalid vote (i.e. a Blank or Null vote) given that the considered agent is a voter.

Let H_v the global field related to the first decision, i.e. to vote (see Fig. S10-b). The ratio of voters, p_v, over registered voters writes as:

$$p_v = \mathcal{P}_>(-H_v), \quad \text{or,} \quad H_v = \ln\left(\frac{p_v}{1-p_v}\right). \tag{S8}$$

(Remind that $p_v = 1 - p_a = p_c + p_{bn}$.) Let $H_{c|v}$ the global bias related to the second binary decision (given that the agent is a voter), that is the bias in favor to put a vote according to the list of choices. The ratio of votes according to the list of choice over voters is written as

$$\frac{p_c}{p_v} = \mathcal{P}_>(-H_{c|v}), \quad \text{or,} \quad H_{c|v} = \ln\left(\frac{\frac{p_c}{p_v}}{1-\frac{p_c}{p_v}}\right) = \ln\left(\frac{p_c}{p_{bn}}\right). \tag{S9}$$

The second decision to put a Blank or Null vote is such that $H_{bn|v} = -H_{c|v}$ (since $p_{bn}/p_v = \mathcal{P}_>(-H_{bn|v})$ and $H_{bn|v} = \ln\left(\frac{p_{bn}}{p_c}\right)$).

According to this two sequential binary choices, the global field which leads a registered voter to put a Valid vote is $H'_c = H_v + H_{c|v} = \ln\left(\frac{p_v \cdot p_c}{p_a \cdot p_{bn}}\right)$; and to put a Blank or Null vote is $H'_{bn} = H_v + H_{bn|v} = \ln\left(\frac{p_v \cdot p_{bn}}{p_a \cdot p_c}\right)$. When Blank or Null ratio is very small ($p_{bn} \ll 1$), $p_c \simeq p_a$, hence one has $H'_{bn} \simeq \tau_3$. So, statistics of H'_{bn} are expected to be very similar to those of τ_3.

If this sequential binary decisions point of view was correct, $H'_{bn} = \ln\left(\frac{p_v \cdot p_{bn}}{p_a \cdot p_c}\right)$ and $H'_c = \ln\left(\frac{p_v \cdot p_c}{p_a \cdot p_{bn}}\right)$ would share the same main features. However, this is strongly rejected by the empirical data. The shape of the distribution over polling stations of the 100 most populated towns of $H'_{bn} - \langle H'_{bn}\rangle$ is not constant from election to election (not shown here), this for each country over various elections. This is confirmed making use of Kolmogorov-Smirnov tests.

To conclude the analysis of the fluctuations around the mean of τ_3, H'_{bn} and H'_c allows one to reject the hypothesis of a sequential binary choice, and to support the one of two mutually exclusive choices.

[13] When one of the three ratios $\{p_a, p_c, p_{bn}\}$ is equal to zero, τ_3 is undefined.

E. Looking for signs of tension, through polling stations analysis

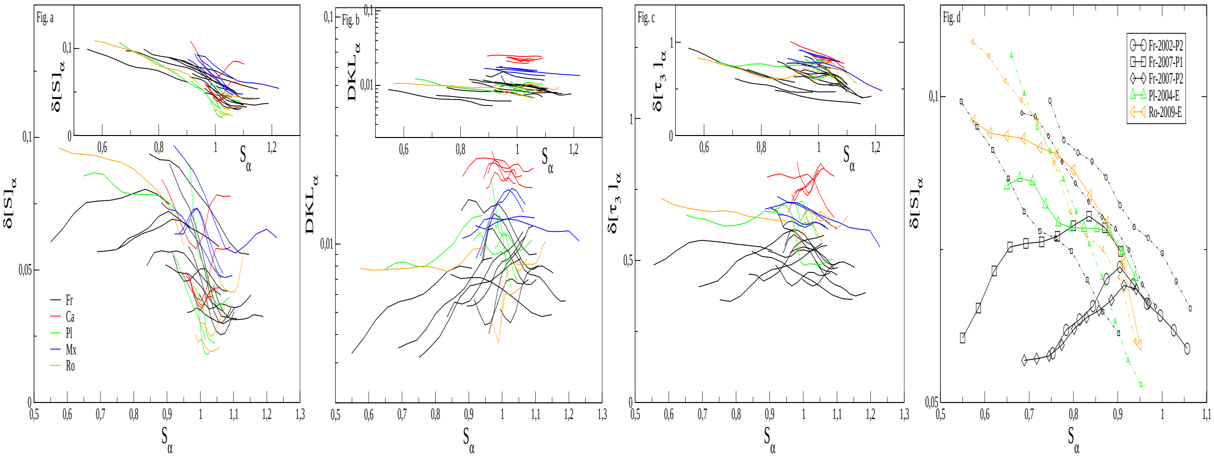

Figure S11: **Civic-involvement heterogeneity (at the polling station scale) in a town with respect to the involvement entropy of the town.** Curves are smoothed and concern the 100 most populated municipalities. Benchmark (see text) curves are plotted in the insets. Heterogeneity measures result from standard deviation of involvement entropy of polling stations (S11-a), KullbackLeibler divergence between a polling stations and other polling stations of the town (S11-b), and standard deviation of logarithmic 3 choices value of polling stations (S11-c). Fig. S11-d: same as Fig. S11-a, but restricted for the 5 elections which deviate the more from $S \approx 1$, where plain lines and dashed line plot respectively real data and benchmark curves.

This section seeks to detect some 'tension', in connection with the involvement entropy. We follow the assumption that 'tension' have some effects for polling stations heterogeneity inside a town. In other words, we try to detect some specific variation of polling stations heterogeneity in a given town, in connection with the involvement entropy of this town. Polling stations (inside a same town) heterogeneity is investigated by three different ways: (1) standard deviation of involvement entropies over all polling stations of the considered town; (2) KullbackLeibler divergence from one polling station compared to other polling station of the town; (3) standard deviation of the logarithmic three choices value (because the shape of its distribution is stable, see the SI Section D) over all polling stations of the town.

The analysis uses polling stations inside the 100 most populated towns (see the SI Section A for more details). Real results will be compared to a benchmark. The benchmark is based on the same heterogeneity of ratios p_a (idem for p_c, and p_{bn}) of polling stations for every town.

Let a town and a polling station of this town respectively called α and α_i. The polling station α_i has some measures, for instance its number of registered voters N_{α_i}, and the set of 3 ratios $\{p_{a,\alpha_i}, p_{c,\alpha_i}, p_{bn,\alpha_i}\}$ that provides its involvement entropy S_{α_i} and its logarithmic three choices value τ_{3,α_i}. The average over all the polling stations of the town (weighted by the number of registered voters, N_{α_i}), gives the corresponding value for the whole town α, e.g. the set of 3 ratios $\{p_{a,\alpha}, p_{c,\alpha}, p_{bn,\alpha}\}$, its involvement entropy S_α, and its logarithmic three choices value $\tau_{3,\alpha}$. The weighted (by the number of registered voters) standard deviation over all the polling stations α_i of the town α is written as $\delta[..]_\alpha$, like for instance $\delta[p_a]_\alpha$, etc., $\delta[S]_\alpha$ and $\delta[\tau_3]_\alpha$. These quantify the heterogeneities within the town α.

Fig. S11-a plots involvement entropy heterogeneity of a town α (i.e. $\delta[S]_\alpha$) with respect to its involvement entropy (i.e. S_α). One should pay attention to the fact that $\delta[S]_\alpha$ going trough a minimum as $S_\alpha \approx 1$ could just be a consequence of $|dS|$ having a minimum near $p_{bn} \approx 0$ and $p_a \approx 0.5$. Hence the

benchmark presented here consists in comparing the empirical data with surrogate ones for which the heterogeneity in p_a, p_c an p_{bn} is the same for all municipalities, up to a binomial noise.

Here, the benchmark forces the same heterogeneity of the set of ratios $\{p_a, p_c, p_{bn}\}$ for every town, but keep their initial value of $\{p_a, p_c, p_{bn}\}$ for the whole town. In other words, let a town α, $\delta[p_a]_\alpha$, $\delta[p_c]_\alpha$ and $\delta[p_{bn}]_\alpha$ have the same values than in other towns; but $\{p_{a,\,\alpha}, p_{c,\,\alpha}, p_{bn,\,\alpha}\}$ are the real values of the town α, measured by the election.

The benchmark is realized as follows. First, we measure for each town α, $p_{a,\,\alpha}$ and $p_{bn,\,\alpha}$; and also $\delta[p_a]_\alpha$ and $\delta[p_{bn}]_\alpha$. The average values of heterogeneities $\delta[p_a]_\alpha$ and $\delta[p_{bn}]_\alpha$ over the 100 considered towns are respectively written as $\overline{\delta_{p_a}}$ and $\overline{\delta_{p_{bn}}}$. Secondly, we drawn from a binomial distribution, for each polling station α_i, its number of registered voters who do not take part to the election ($N_{a,\,\alpha_i}$) and the number of Blank and Null votes ($N_{bn,\,\alpha_i}$), such that:

$$\begin{aligned} N_{a,\,\alpha_i} &= \mathcal{B}(N_{\alpha_i}\ ;\ p_{a,\,\alpha} + \eta_a), \\ N_{bn,\,\alpha_i} &= \mathcal{B}(N_{\alpha_i}\ ;\ p_{bn,\,\alpha} + \eta_{bn}), \end{aligned} \tag{S10}$$

where η_a and η_{bn} are independent Gaussian noises of mean 0 and of standard deviation $\overline{\delta_{p_a}}$ and $\overline{\delta_{p_{bn}}}$ respectively, and N_{α_i} is the real number of registered voters of the polling station α_i of the considered town α. Note that we use a binomial distribution in order to take into account finite size effects; and here, for each citizen in a surrogate-polling station, probabilities to not vote and to put a null-blank vote are mutually independent.

Instead of making use of standard-errors, an alternative measure of heterogeneity is provided by making use of the so-called KullbackLeibler divergence which characterizes the difference between two probability distributions. For each polling station α_i of a given town α, we compute the divergence DKL_{α_i} from the polling station distribution P_{α_i} to the rest of the town, $Q_{\alpha_i} \equiv P_{\alpha-\alpha_i}$,

$$DKL_{\alpha_i} \equiv \sum_j P_{\alpha_i}(j) \log \frac{P_{\alpha_i}(j)}{Q_{\alpha_i}(j)} \tag{S11}$$

where, here and in the following, for any distribution we write $P(j), j = 1, 2, 3,$ instead of p_a, p_c, p_{bn}. Then we compute the mean KullbackLeibler divergence, DKL_α, of the town α by averaging over all polling stations, weighting by the corresponding number of registered voters, N_{α_i}.

$$DKL_\alpha \equiv \sum_i \frac{N_{\alpha_i}}{N_\alpha}\, DKL_{\alpha_i} \tag{S12}$$

This mean KullbackLeibler divergence DKL_α gives us a measure of heterogeneity of polling stations into a town α.

Fig. S11 compares benchmarks curves and empirical data [14]. It appears that, the smaller the involvement entropy S (with $S \lesssim 0.85$), the smaller the involvement entropy heterogeneity at the polling station level (see more specifically Fig. S11-d). Heterogeneity of polling stations in a same town is measured via three different ways: standard deviations of the involvement entropy and the logarithmic three choices ratio, and also via the KullbackLeibler divergence. In other words, the more the town is “ordered” (for its electorate civic-involvement), the more the town is homogeneous (at the polling station scale, and still for a civic involvement point of view). Note also that this point is particularly clear when the ratio p_c is high (e.g. for 3 French elections), compared to cases where p_a are high (e.g. for European Parliament elections in Romania and Poland). It can also be noted that often heterogeneity of involvement entropies of polling stations inside towns ($\delta[S]_\alpha$) has a significantly minimal value when their involvement entropies (S_α) are around 1, and this minimization is much more marked for real data than for benchmark ones.

[14] In this section, extreme values greater than *3 sigma* are note taken into account in order to remove some electoral errors, etc.

F. Disentangling Blank votes from Null votes

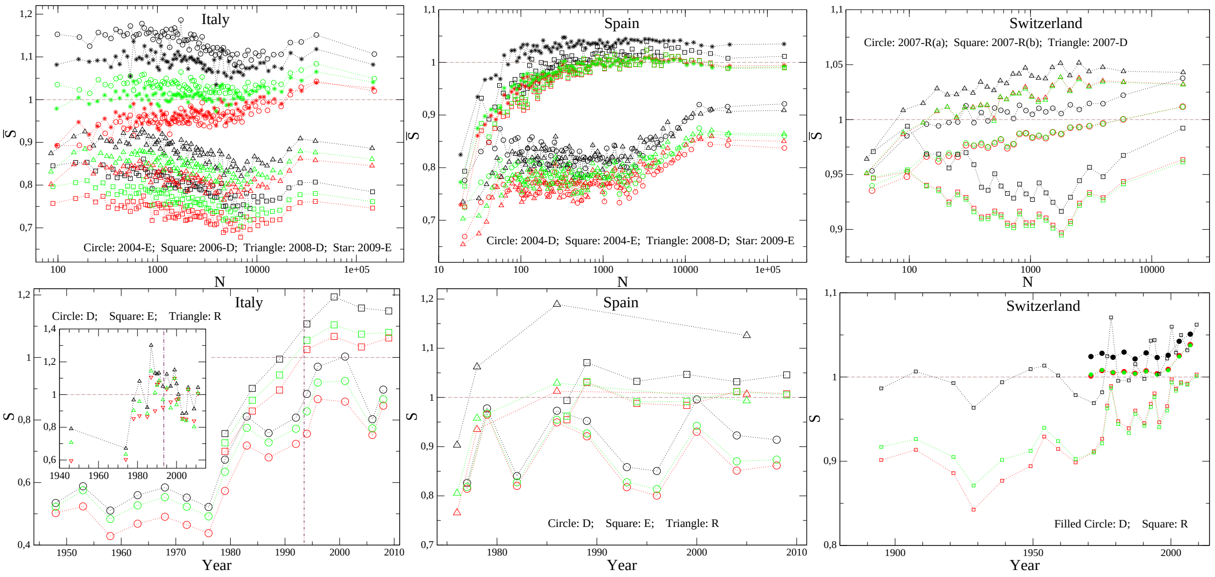

Figure S12: Blank votes are grouped with: (1) Null votes (like in the main text, cf. Eq. (2)) in black; (2) Valid Votes or another vote included in the list of choices (cf. Eq. (S14)) in red; (3) citizens who do not take part to the election (cf. Eq. (S15)) in green. Top: mean values of S, $S_{b\equiv c}$, $S_{b\equiv a}$, over bins with around 100 municipalities of size $\approx N$ (like in Fig. 5). Bottom: Evolution in time of S, $S_{b\equiv c}$, $S_{b\equiv a}$ (with the same scale of aggregate data as in Figs. 6 and S1). For the sake of clarity, standard deviations over Swiss *Cantons* and Spanish *Comunidades autónomas* are note shown. Each point (R) for Swiss graph gives the average of around 20 Swiss referendums. The end of Italian compulsory voting is shown by a vertical line.

Italy [15], Spain and Switzerland [16] are countries for which Blank votes are distinguished from Null votes. Let N_b and N_n the number of citizens who respectively vote Blank and Null amongst N registered voters (of one municipality, *Canton*, *Comunidad autónoma*, the whole country). Ratios, or probabilities, to respectively vote Blank and Null are

$$p_b = \frac{N_b}{N}, \quad p_n = \frac{N_n}{N}. \tag{S13}$$

In such cases, it is legitimate to consider that Blank votes should be categorized with votes in favor of one of a the proposed choices to the election. Then, the Blank vote has not a 'marginal' involvement meaning, like previously, but its citizen involvement is similar to another Valid vote according to the list of choices of the election. One should then consider a modified involvement entropy, defined from the 3-set ratios (of sum unity) $\{p_a, (p_c + p_b), p_n\}$, that is

$$S_{b\equiv c} = -p_a \log(p_a) - (p_c + p_b)\log(p_c + p_b) - p_n \log(p_n). \tag{S14}$$

Alternatively, one may consider that Blank votes loose their 'marginal' aspect in citizen involvement, and should be categorized as votes from citizen who do not take part to the election. Then the relevant

[15] We only analyze the first question asked in a Referendum. Senate elections are note shown in Fig. S12-below because they are very similar to Chamber of Deputies (D) elections.

[16] Chamber of deputies elections (D) distinguish, in our database, Blank vote between Null votes since 1971; and since 1887 for *votations* (or referendums).

modified involvement entropy, defined from the 3-set ratios (still of sum unity) $\{(p_a + p_b), p_c, p_n\}$, writes as

$$S_{b\equiv a} = -(p_a + p_b)\log(p_a + p_b) - p_c \log(p_c) - p_n \log(p_n). \tag{S15}$$

Figures S12 shows for Italy, Spain and Switzerland, the involvement entropy, S, and the modified versions, $S_{b\equiv c}$ and $S_{b\equiv a}$: (1) for municipalities and with respect to the municipality-size N (as in Fig. 5); (2) for the whole country (directly for Italy, and as a weighted mean by population-size over 25 or 26 Swiss *Cantons* and 17 or 19 Spanish *Comunidades autónomas*) as a function of time (as in Figs. 6 and S1). Fig. 13 shows the modified involvement entropy $S_{b\equiv c}$ ($S_{b\equiv a}$ which is not shown, is very close to $S_{b\equiv c}$), with respect to the involvement entropy S, for ~ 530 Swiss Referendums.

Figure S12 exhibits some trends and regularities that depend on the values of involvement entropy S. (1) When $S < 1$ (e.g. in Italian and Spanish Chamber of Deputies elections, both at municipality scale or at large scale of aggregate data), modified involvement entropies are smaller than S. This means a greater order of the modified citizen involvement. It can be interpreted as follows: the loss of nuance or specificity (for citizen involvement) that Blank vote have, implies a greater polarization or heterogeneity of the electorate, still split into 3 groups. (2) When $S \approx 1$, two different cases arise. First, for Spanish European Parliament elections, Swiss Chamber of Deputies elections and Referendums (uniquely for the latter, since the 2000s), both at municipality scale or at large scale of aggregate data: the modified involvement entropies are slightly lower than S, but still ≈ 1. Second, for earlier Swiss Referendums, and particularly before the 1960s: $S_{b\equiv c}$ (or $S_{b\equiv a}$) are lower than S, but not slightly lower. (3) When $S > 1$ and $S \not\approx 1$ (e.g. for Italian European Parliament elections, both at municipality scale or at large scale of aggregate data, and Spanish Referendums, particularly 1986 and 2005 ones, at provincial scale), modified involvement entropies are still lower than S. But one more time, it is surprising to notice that modified involvement entropies are such that $S_{b\equiv c} \approx 1$ (or $S_{b\equiv a} \approx 1$). It can be explained as follows: subtlety or specificity of citizen involvement due to Blank votes means an increasing of disorder of the electorate involvement. The loss of this subtlety or specificity (i.e. considering Blank votes like another vote in favor of the list of choices, or like another abstentionist) implies a loss of 'tension' contained in electoral campaign. And strikingly, this loss of 'tension' provides a new entropy, like the usual common-value of involvement entropy, ≈ 1.

Note that above items (1) and (3) (i.e. when significantly $S < 1$ or $S > 1$), pointed out in Fig. S12, are clearly shown in Fig. 13 for Swiss Referendums. (In the Fig. 13, S' means $S_{b\equiv c}$, which is very near to $S_{b\equiv a}$ on average.) Note also that the surprising plateau (which provides modified involvement entropies equal to ≈ 1, on average, when $S \gtrsim 1.05$) does not exist, in our database, for most populated municipalities. For the latter case (not shown), the around 100 most populated municipalities for which $S \gtrsim 1.05$, uniquely provides $S_{b\equiv c}$ (or $S_{b\equiv a}$) lower than S such that, on average, $\overline{S_{b\equiv c}} \approx 1$ (or $\overline{S_{b\equiv a}} \approx 1$), but without a plateau.

Lastly, this study does not allow us to know whether it is more meaningful (according to the entropy of the electorate involvement) to consider Blank vote like another vote proposed in the list of choices or like another abstentionist vote. Nevertheless, in our database, Blank votes seem more meaningful that Null Votes in Spain and in Switzerland. Indeed, when p_n and p_b are interchanged between each other in Eqs. (S14) or (S15), above item (3) (when significantly $S > 1$, then the modified involvement entropy is ≈ 1) does clearly not exist for Spanish and Swiss Referendums.

To conclude, let us recall the main point of this section: when involvement entropy S does not obey to the common occurrence $S \approx 1$ for high population-size municipalities, or at large aggregate scale, because the citizen involvement of the electorate is too much disordered (i.e. significantly $S > 1$), then the modified involvement entropy (by the loss of the specificity of Blank votes) takes on average the same common value ≈ 1.

References

1. http://www.bmi.gv.at/cms/BMI_wahlen/
   http://sunsite.univie.ac.at/Austria/elections/

2. http://www.elections.ca/content.aspx?section=ele&dir=pas&document=index&lang=f

3. http://www.czso.cz

4. http://www.interieur.gouv.fr/sections/a_votre_service/elections/resultats

5. *Amt für Statistik Berlin-Brandenburg*, DVD: Statistik lokal, Ausgabe 2004, 2006, 2010

6. http://www.interno.it

7. http://www.ife.org.mx/documentos/RESELEC/SICEEF/principal.html
   http://www.ife.org.mx/portal/site/ifev2/Estadisticas_y_Resultados_Electorales/

8. http://www.pkw.gov.pl/wybory2000/gminy/index.html
   http://www.pkw.gov.pl/katalog/artykul/17706.html
   http://referendum.pkw.gov.pl/arkusze/index.html
   http://www.pe2004.pkw.gov.pl/
   http://www.pkw.gov.pl/pkw2/index.jsp?place=Menu01&news_cat_id=1818&layout=1
   http://www.pkw.gov.pl/pkw2/index.jsp?place=Menu01&news_cat_id=1819&layout=1
   http://wybory2007.pkw.gov.pl/SJM/EN/WYN/W/index.htm
   http://pe2009.pkw.gov.pl/PUE/EN/WYN/W/index.htm
   http://prezydent2010.pkw.gov.pl/PZT1/EN/WYN/W/index.htm
   http://prezydent2010.pkw.gov.pl/PZT/EN/WYN/W/index.htm

9. http://www.bec2009pe.ro/rezultate.html
   http://www.bec2009p.ro/rezultate.html

10. http://www.infoelectoral.mir.es/min/

11. http://www.bfs.admin.ch

12. http://www.bmi.gv.at/cms/BMI_wahlen/

13. http://www.elections.ca/content.asp?section=pas&document=turnout&lang=f&textonly=false
    http://www.elections.ca/content.asp?section=gen&document=appx&dir=his&lang=f&textonly=false

14. http://www.volby.cz/index_en.htm
    http://www2.essex.ac.uk/elect/database/indexCountry.asp?

15. Database EDEN, Jean Chiche, CEVIPOF
    http://mjp.univ-perp.fr/france/ref.htm

16. http://www.bundeswahlleiter.de/

17. http://elezionistorico.interno.it/index.php

18. http://www.ife.org.mx/documentos/RESELEC/SICEEF/principal.html

19. http://www2.essex.ac.uk/elect/database/indexCountry.asp? http://www.pkw.gov.pl/

20. http://www2.essex.ac.uk/elect/database/indexCountry.asp?
www.bec2004.ro www.becreferendum2007.ro www.becreferendum2007vu.ro
http://alegerileeuropene.blogspot.com/2007/11/biroul-electoral-central-bec-rezultate.html
www.becparlamentare2008.ro www.bec2009pe.ro
www.bec2009p.ro, http://alegeri.referinte.transindex.ro/

21. http://www.infoelectoral.mir.es/min/

22. http://www.bfs.admin.ch/bfs/portal/fr/index/themen/17/02/blank/data/04/01.html
http://www.admin.ch/ch/f/pore/va/vab_2_2_4_1.html

23. http://www.u-cergy.fr/fr/laboratoires/labo-lptm/donnees-de-recherche.html

24. C. Borghesi, *Une étude de physique sur les élections – Régularités, prédictions et modèles sur des élections françaises*, Ed. universitaires européennes, 2009

25. C. Borghesi and J.-P. Bouchaud, *Spatial correlations in vote statistics: a diffusive field model for decision-making*, Eur. Phys. J. B **75**, 395-404 (2010)

26. S. P. Anderson, A de Palma, J.-F. Thisse, *Discrete Choice Theory of Product Differentiation*, MIT Press, 1992

27. C. Borghesi, J.-C. Raynal and J.-P. Bouchaud, *Election Turnout Statistics in Many Countries: Similarities, Differences, and a Diffusive Field Model for Decision-Making*, PLoS ONE 7(5): e36289 (2012)

28. C. Borghesi, *Three quantitative predictions based on past regularities about voter turnout at the French 2009 European election*, arXiv:0905.4578 (2009)

29. C. Borghesi, *Results of three quantitative predictions based on past regularities about voter turnout at the French 2009 European election*, arXiv:0906.2692 (2009)
