# Supplementary material for: Between Order and Disorder: A ‘Weak Law’ on Recent Electoral Behavior among Urban Voters?
Source: PLoS One. 2012 Jul 25;7(7):e39916. doi: 10.1371/journal.pone.0039916 (PMC3405122; doi:10.1371/journal.pone.0039916)
Supplement: Table S1 — Elections studied in this paper at the municipality scale. See Appendix S1, Section A, for more details. (PDF) [file pone.0039916.s008.pdf]

Id	$\overline{S}$	$\overline{p_a}$	$\overline{p_c}$	$\overline{p_{bn}}(\overline{p_b})$	Id	$\overline{S}$	$\overline{p_a}$	$\overline{p_c}$	$\overline{p_{bn}}(\overline{p_b})$
Fr 1992 R	**1.02**±0.04	0.32	0.66	0.018	Fr 1993 D	1.09±0.04	0.34	0.63	0.028
Fr 1994 E	1.12±0.03	0.48	0.50	0.020	Fr 1995 P1	0.91±0.04	0.24	0.74	0.018
Fr 1995 P2	**1.01**±0.07	0.23	0.73	0.044	Fr 1997 D	**1.08**±0.03	0.36	0.62	0.024
Fr 1998 rg	1.11±0.03	0.46	0.52	0.019	Fr 1999 E	1.11±0.03	0.54	0.44	0.020
Fr 2000 R	**1.02**±0.07	0.71	0.25	0.036	Fr 2002 P1	**1.01**±0.04	0.31	0.67	0.019
Fr 2002 P2	0.95±0.07	0.21	0.75	0.035	Fr 2002 D	**1.02**±0.04	0.37	0.62	0.010
Fr 2004 rg	1.10±0.04	0.41	0.57	0.021	Fr 2004 E	**1.04**±0.03	0.57	0.42	0.010
Fr 2005 R	**1.00**±0.05	0.32	0.66	0.014	Fr 2007 P1	0.72±0.08	0.17	0.82	0.010
Fr 2007 P2	0.84±0.06	0.17	0.80	0.032	Fr 2007 D	**1.04**±0.03	0.42	0.57	0.009
Fr 2009 E	**1.03**±0.05	0.60	0.39	0.012	Fr 2010 rg	**1.06**±0.03	0.57	0.42	0.012
At 1994 D	0.81±0.11	0.20	0.78	0.016	At 1995 D	0.73±0.10	0.15	0.83	0.018
At 1996 E	**1.04**±0.04	0.33	0.65	0.021	At 1998 P	**1.00**±0.10	0.27	0.70	0.032
At 1999 E	**1.06**±0.05	0.52	0.46	0.013	At 1999 D	0.82±0.09	0.22	0.77	0.011
At 2002 D	0.73±0.10	0.17	0.81	0.011	At 2004 P	**1.04**±0.09	0.31	0.66	0.028
At 2004 E	**1.03**±0.05	0.59	0.40	0.010	At 2006 D	0.87±0.09	0.24	0.74	0.012
At 2008 D	0.88±0.08	0.24	0.75	0.014	At 2009 E	**1.04**±0.04	0.55	0.44	0.009
At 2010 P	1.16±0.06	0.48	0.49	0.034					
Pl 2000 P1	**0.98**±0.03	0.36	0.63	0.006	Pl 2001 D	1.09±0.02	0.52	0.46	0.015
Pl 2003 R	**0.98**±0.02	0.37	0.62	0.004	Pl 2004 E	0.79±0.07	0.78	0.22	0.005
Pl 2005 D	**1.06**±0.03	0.58	0.41	0.013	Pl 2005 P1	**1.02**±0.01	0.49	0.51	0.003
Pl 2005 P2	**1.03**±0.01	0.47	0.53	0.006	Pl 2007 D	**1.05**±0.03	0.42	0.57	0.010
Pl 2009 E	0.87±0.06	0.73	0.27	0.004	Pl 2010 P1	**1.01**±0.02	0.43	0.57	0.004
Pl 2010 P2	**1.03**±0.02	0.43	0.56	0.007					
Ge 2002 D	0.83±0.07	0.22	0.77	0.009	Ge 2004 Ld	**1.02**±0.04	0.41	0.58	0.007
Ge 2004 E	**1.02**±0.05	0.59	0.40	0.009	Ge 2005 D	0.87±0.06	0.24	0.75	0.011
Ge 2009 E	**1.00**±0.05	0.60	0.40	0.006	Ge 2009 D	0.95±0.05	0.30	0.69	0.009
Ge 2010 Ld	**1.04**±0.03	0.43	0.56	0.009					
Ca 1997 D	**1.00**±0.04	0.37	0.62	0.009	Ca 2000 D	**1.03**±0.03	0.44	0.56	0.006
Ca 2004 D	**1.02**±0.02	0.46	0.54	0.004	Ca 2006 D	**1.01**±0.02	0.44	0.56	0.003
Ca 2008 D	**1.02**±0.02	0.49	0.51	0.003					
It 2004 E	1.11±0.12	0.29	0.66	0.053(0.023)	It 2006 D	0.78±0.13	0.17	0.81	0.020(0.007)
It 2008 D	0.89±0.12	0.20	0.77	0.027(0.008)	It 2009 E	1.08±0.10	0.36	0.61	0.034(0.013)
Mx 2003 D	**1.04**±0.05	0.59	0.40	0.013	Mx 2006 D	**1.04**±0.04	0.40	0.58	0.012
Mx 2006 P	**1.03**±0.04	0.40	0.59	0.010	Mx 2009 D	1.11±0.06	0.56	0.41	0.027
Ro 2009 E	0.73±0.09	0.81	0.18	0.008	Ro 2009 R	1.09±0.02	0.55	0.44	0.017
Ro 2009 P1	**1.05**±0.02	0.52	0.48	0.008	Ro 2009 P2	**1.04**±0.02	0.50	0.50	0.006
Sp 2004 D	0.92±0.07	0.24	0.74	0.020(0.014)	Sp 2004 E	**1.01**±0.06	0.57	0.42	0.006(0.003)
Sp 2008 D	0.91±0.08	0.26	0.73	0.013(0.009)	Sp 2009 E	**1.03**±0.04	0.56	0.43	0.009(0.006)
CH 2007 R(a)	**1.04**±0.04	0.53	0.46	0.008(0.004)	CH 2007 R(b)	**0.99**±0.06	0.62	0.37	0.007(0.004)
CH 2007 D	**1.04**±0.05	0.53	0.47	0.009(0.002)					
Cz 2003 R	**1.07**±0.01	0.47	0.52	0.012					

Table S1: **Elections studied in this paper at the municipality scale.** An election is identified (Id) by its country, its year date and its nature. D: Chamber of Deputies election; E: European parliament election; P: presidential election (according to the constitution of the country, in only one round); P1 and P2: first and second round of a Presidential election; R: Referendum; Ld: German *Länder* elections; rg: French *Régionales* elections. For each country elections are given in a chronological order (but the 2006 Mexican Presidential (P) and Deputies (D) elections occurred the same day, and also for the 2009 Romanian Presidential (P1) and Referendum (R) elections). Even if an election needs two rounds, only the first one is considered (e.g. the French Deputies (D) and *Régionales* (rg) elections) unless the contrary is indicated (e.g. P1 and P2). Mean values of S, p_a, p_c, p_{bn}(and (p_b) if Blank Vote are distinguished between Null Vote), and also standard deviation only for S, are given over the bin of the $\approx$ 100 (or $\approx$ 200 for France only) most populated municipalities. In bold text, $\overline{S} \in [0.98; 1.08]$. See Appendix S1, Section A, for more explanation.
