# Supplementary material for: Between Order and Disorder: A ‘Weak Law’ on Recent Electoral Behavior among Urban Voters?
Source: PLoS One. 2012 Jul 25;7(7):e39916. doi: 10.1371/journal.pone.0039916 (PMC3405122; doi:10.1371/journal.pone.0039916)
Supplement: Table S2 — Elections studied in this paper at large scale (national, provincial, etc.) for their evolution in time. See Appendix S1, Section A, for more details. (PDF) [file pone.0039916.s009.pdf]

Country	Kind of elections	Scale of aggregate data
At	D, E, P, R	National
Ca	D	Province (5-13)
CH	D, R	*Canton* (25-26)
Cz	D, E, R, rg, S1, S2	National
Fr	Cant, D, E, P1, P2, R, rg	*département* (90-96)
Ge	D, E	*Land* (9-16)
It	D, E, R, S	National
Mx	D, P	National
Pl	D, E, P1, P2	National
Ro	D, E, P1, P2, R	National
Sp	D, E, R	*Comunidad autónoma* (17-19)

Table S2: **Elections studied in this paper at large scale for their evolution in time.** Notation is the same as in Tab. S1. For Czech Republic, "rg" means Election into regional councils, "S1" and "S2" are respectively the first and second round of the Senate elections; for France, "Cant" refers to the *Cantonales* elections and some referendums are only known at the national scale; for Italy, "S" means Senate elections, and occur at the same time as Deputies elections (D) but with older registered voters. In parenthesis, the total number of different provinces (or *Cantons*, etc.), which can change in time, in the whole country. See Appendix S1, Section A, for more explanation.
